# Cohort profile: the CORDELIA study (*Collaborative cOhorts Reassembled Data to study mEchanisms and Longterm Incidence of chronic diseAses*)

**DOI:** 10.1007/s10654-025-01229-6

**Published:** 2025-05-12

**Authors:** Álvaro Hernáez, Anna Camps-Vilaró, Sara Polo-Alonso, Isaac Subirana, Rafel Ramos, Rafael de Cid, Fernando Rodríguez-Artalejo, Roberto Elosua, M. Dolores Chirlaque, Pilar Amiano, Marcelino Bermúdez-López, Marcela Guevara, Sergio Cinza-Sanjurjo, María-José Sánchez, Antonio Cabrera de León, Martín Laclaustra, Gemma Rojo-Martínez, María J. Guembe-Suescun, Beatriz Pérez-Gómez, Tomás Vega-Alonso, Pere Torán-Monserrat, David Lora-Pablos, José María Huerta, José M. Valdivielso, Irene R. Dégano, Francisco J. Félix-Redondo, Ana María Gandarillas, Sergio Valdés, Xavier Mundet-Tuduri, Pedro L. Sánchez, Vicente Martín-Sánchez, Fernando Rigo, Manuela Alonso-Sampedro, Conchi Moreno-Iribas, Juan Carlos Martín-Escudero, Elías Delgado, Maria Grau, Inés Urrutia, Diana Ovejero, Inés Quintela, Ruth Martí-Lluch, Natalia Blay, José R. Banegas, Helena Tizón-Marcos, Jesús Humberto Gómez, Amaia Aizpurua, Eva Castro-Boqué, Josu Delfrade, Miguel Ángel Prieto-Díaz, Miguel Rodríguez-Barranco, Delia Almeida-González, Belén Moreno-Franco, Wasima Oualla-Bachiri, Carmen Sayón-Orea, Elena Plans-Beriso, José Eugenio Lozano, Víctor M. López-Lifante, Pilar Cancelas-Navia, Natalia Cabrera-Castro, Serafí Cambray, Lluís Zacarías-Pons, Daniel Fernández-Bergés, Encarnación Donoso-Navarro, Cristina Maldonado-Araque, Josep Franch-Nadal, Pedro Ignacio Dorado-Díaz, Alejandro Villarín-Castro, Guillem Frontera-Juan, Francisco Gude, Naroa Andueza, María Téllez-Plaza, Jessica Ares-Blanco, Raquel Cruz, Marc Ribas-Aulinas, Jordi Barretina, Pilar Guallar-Castillón, Miguel Caínzos-Achirica, Sandra Milena Colorado-Yohar, Adrián Llorente, Juan Miguel Diaz-Tocados, Eva Ardanaz, Rafael Manuel Micó-Pérez, Nicolás Francisco Fernandez-Martinez, María del Cristo Rodríguez-Pérez, Ana Cenarro, Alfonso L. Calle-Pascual, Jaume Marrugat

**Affiliations:** 1 REGICOR Study Group, Hospital del Mar Research Institute (IMIM), Carrer Doctor Aiguader 88, 08003 Barcelona, Spain; 2 CIBER de Enfermedades Cardiovasculares (CIBERCV), Instituto de Salud Carlos III, Madrid, Spain; 3 Blanquerna School of Health Sciences, University Ramon Llull, Barcelona, Spain; 4 School of Medicine, University of Vic-Central University of Catalonia (UVic-UCC), Vic, Spain; 5 PhD Program, Barcelona, Spain; 6 Cardiovascular Epidemiology and Genetics Research Group, Hospital del Mar Research Institute (IMIM), Barcelona, Spain; 7 Department of Medical Science, Faculty of Medicine, University of Girona (UdG), Girona, Spain; 8 Vascular Health Research Group, Institut Universitari Per a La Recerca en Atenció Primària de Salut Jordi Gol I Gurina (IDIAPJGol), Girona, Spain; 9 Girona Biomedical Research Institute (IDIBGI), Doctor Trueta University Hospital, Girona, Spain; 10 Network for Research On Chronicity, Primary Care, and Health Promotion (RICAPPS), Instituto de Salud Carlos III, Madrid, Spain; 11 Genomes For Life-GCAT Lab, CORE Program, Germans Trias I Pujol Research Institute (IGTP), Badalona, Spain; 12 Grup de Recerca en Impacte de les Malalties Cròniques I les Seves Trajectòries (GRIMTRA), Germans Trias I Pujol Research Institute (IGTP), Badalona, Spain; 13 Department of Preventive Medicine and Public Health, School of Medicine, Universidad Autónoma de Madrid, Madrid, Spain; 14 IMDEA/Food Institute. CEI UAM+CSIC, Madrid, Spain; 15 CIBER de Epidemiología y Salud Pública (CIBERESP), Instituto de Salud Carlos III, Madrid, Spain; 16 Department of Epidemiology, Regional Health Council, IMIB-Arrixaca, Murcia, Spain; 17 Department of Health and Social Sciences, University of Murcia, Murcia, Spain; 18 Sub Directorate for Public Health and Addictions of Gipuzkoa, Ministry of Health of the Basque Government, San Sebastian, Spain; 19 Epidemiology of Chronic and Communicable Diseases Group, BioGipuzkoa Health Research Institute, San Sebastián, Spain; 20 Vascular and Renal Translational Research Group, Institut de Recerca Biomèdica de Lleida (IRBLleida), Lleida, Spain; 21 Department of Experimental Medicine, University of Lleida, Lleida, Spain; 22 Instituto de Salud Pública y Laboral de Navarra, Pamplona, Spain; 23 Navarra Institute for Health Research (IdiSNA), Pamplona, Spain; 24 Milladoiro Health Centre, Health Area of Santiago de Compostela, Santiago de Compostela, Spain; 25 Research Methods Group (RESMET), Health Research Institute of Santiago de Compostela (IDIS), Santiago de Compostela, Spain; 26 Department of Medicine, University Hospital of Santiago de Compostela, Santiago de Compostela, Spain; 27 Escuela Andaluza de Salud Pública, Granada, Spain; 28 Instituto de Investigación Biosanitaria Ibs.GRANADA, Granada, Spain; 29 University Hospital Nuestra Señora de Candelaria, Santa Cruz de Tenerife, Spain; 30 Department of Preventive Medicine, Faculty of Medicine, University of la Laguna, San Cristóbal de La Laguna, Spain; 31 Instituto de Investigación Sanitaria de Aragón (IIS Aragón), Saragossa, Spain; 32 Translational Research Unit, Hospital Universitario Miguel Servet, Saragossa, Spain; 33 Faculty of Medicine, Universidad de Zaragoza, Saragossa, Spain; 34 Department of Endocrinology and Nutrition, Hospital Regional Universitario de Málaga, Universidad de Málaga, Málaga, Spain; 35 Instituto de Investigación Biomedica de Málaga (IBIMA-Plataforma Bionand), Málaga, Spain; 36 CIBER de Diabetes y Enfermedades Metabólicas Asociadas (CIBERDEM), Instituto de Salud Carlos III, Madrid, Spain; 37 Vascular Risk in Navarre Investigation Group, Department of Health, Government of Navarre, Pamplona, Spain; 38 Servicio de Apoyo a la Gestión Clínica y Continuidad Asistencial, Hospital Universitario de Navarra, Pamplona, Spain; 39 Department of Chronic Diseases, National Center of Epidemiology, Instituto de Salud Carlos III, Madrid, Spain; 40 Dirección General de Salud Pública, Consejería de Sanidad, Junta de Castilla y León, Valladolid, Spain; 41 Unitat de Suport a la Recerca Metropolitana Nord, Institut Universitari Per a la Recerca a L’Atenció Primària de Salut Jordi Gol I Gurina (IDIAPJGol), Mataró, Spain; 42 Multidisciplinary Research Group in Health and Society (GREMAS), Institut Universitari Per a la Recerca en Atenció Primària de Salut Jordi Gol I Gurina (IDIAPJGol), Barcelona, Spain; 43 Germans Trias I Pujol Research Institute, Can Ruti Campus, Badalona, Spain; 44 Instituto de Investigación Sanitaria del Hospital Universitario, 12 de Octubre (imas12), Madrid, Spain; 45 Spanish Clinical Research Network (SCReN), Madrid, Spain; 46 Facultad de Estudios Estadísticos, Universidad Complutense de Madrid, Madrid, Spain; 47 Department of Medicine and Surgery, University of Lleida, Lleida, Spain; 48 Institute for Research and Innovation in Life Sciences and Health in Central Catalonia (IRIS-CC), Vic, Spain; 49 Dirección General de Asistencia Sanitaria. Servicio Extremeño de Salud, Vic, Spain; 50 Research Unit of Don Benito-Villanueva de La Serena Health Area, Servicio Extremeño de Salud. Villanueva de La Serena, Badajoz, Spain; 51 University Institute for Biosanitary Research of Extremadura (INUBE), Badajoz, Spain; 52 Subdirección General de Vigilancia en Salud Pública, Dirección General de Salud Pública, Consejería de Sanidad, Madrid, Spain; 53 Unitat de Suport a la Recerca Barcelona, Institut Universitari Per a la Recerca a L’Atenció Primària de Salut Jordi Gol I Gurina (IDIAPJGol), Barcelona, Spain; 54 Complejo Asistencial Universitario de Salamanca, Salamanca, Spain; 55 Instituto de Investigación Biomédica de Salamanca (IBSAL), Universidad de Salamanca, Salamanca, Spain; 56 Instituto de Biomedicina (IBIOMED), Universidad de León, León, Spain; 57 Hospital Universitari Son Espases Atención Primaria Mallorca, Institut d’Investigació Sanitària Illes Balears (IdISBa), Palma, Spain; 58 Department of Internal Medicine, Hospital Universitario Rio Hortega, University of Valladolid, Valladolid, Spain; 59 Endocrinology, Nutrition, Diabetes and Obesity Group, Health Research Institute of the Principality of Asturias (ISPA), Oviedo, Spain; 60 Asturias Central University Hospital, Oviedo, Spain; 61 Department of Medicine, University of Oviedo, Oviedo, Spain; 62 CIBER de Enfermedades Raras (CIBERER), Instituto de Salud Carlos III, Madrid, Spain; 63 Department of Medicine, School of Medicine and Health Sciences, Serra-Húnter Fellow, University of Barcelona, Barcelona, Spain; 64 August Pi I Sunyer Biomedical Research Institute (IDIBAPS), Barcelona, Spain; 65 Instituto de Investigación Sanitaria Biocruces, Hospital Universitario de Cruces, Barakaldo, Spain; 66 European Reference Network On Rare Endocrine Conditions (Endo-ERN), Leiden, The Netherlands; 67 Universidad del País Vasco/Euskal Herriko Unibertsitatea (UPV/EHU), Leioa, Spain; 68 Musculo-Skeletal Research Unit, Hospital del Mar Research Institute (IMIM), Barcelona, Spain; 69 CIBER de Fragilidad y Envejecimiento Saludable (CIBERFES), Instituto de Salud Carlos III, Madrid, Spain; 70 Fundación Pública Galega de Medicina Xenómica, Centro Nacional de Genotipado (CEGEN), Santiago de Compostela, Spain; 71 Cardiology Service, Hospital del Mar, Barcelona, Spain; 72 Vallobín-La Florida Health Center, Principality of Asturias Health Service, Oviedo, Spain; 73 CIBER de Fisiopatología de La Obesidad y Nutrición (CIBEROBN), Instituto de Salud Carlos III, Madrid, Spain; 74 Department of Preventive Medicine and Public Health, University of Navarra, Pamplona, Spain; 75 Primary Healthcare Centre Palau Solità I Plegamans, Gerència d’Atenció Primària Metropolitana Nord, Institut Català de la Salut, Barcelona, Spain; 76 Department of Basic Medical Sciences, Serra Húnter Lecturer, University of Lleida, Lleida, Spain; 77 Department of Biochemistry-Clinical Analysis, Puerta de Hierro Majadahonda University Hospital, Madrid, Spain; 78 Unidad Docente Multiprofesional de Atención Familiar y Comunitaria de Toledo, Gerencia de Atención Primaria de Toledo, Servicio de Salud de Castilla-La Mancha, Toledo, Spain; 79 Faculty of Medicine, University of Santiago de Compostela, Santiago de Compostela, Spain; 80 Centro Singular de Investigación en Medicina Molecular y Enfermedades Crónicas (CIMUS), Universidade de Santiago de Compostela, Santiago de Compostela, Spain; 81 Research Group On Demography and Health, National Faculty of Public Health, University of Antioquia, Medellin, Colombia; 82 Xàtiva–Ontinyent Department of Health, Fontanars dels Alforins Health Centre, Valencia, Spain; 83 Instituto Aragonés de Ciencias de la Salud (IACS), Saragossa, Spain; 84 Endocrinology and Nutrition Department, Hospital Clínico Universitario San Carlos, Instituto de Investigación Sanitaria del Hospital Clínico San Carlos (IdISSC), Madrid, Spain; 85 Facultad de Medicina, Medicina II Department, Universidad Complutense de Madrid, Madrid, Spain

**Keywords:** Cohort, Genome-wide association study, Cardiovascular disease, Mortality, Southern Europe

## Abstract

The CORDELIA Study (Collaborative Cohorts Reassembled Data to Study Mechanisms and Long-term Incidence of Chronic Diseases) combines 35 Spanish population cohorts to investigate the clinical, environmental, genetic, and omics determinants of cardiovascular disease in the Southern European population. It aims to conduct the largest genome-wide association study to date on cardiovascular disease in this population, improve predictions of cardiovascular incidence using genomic and clinical data, and identify subgroups that would benefit most from targeted pharmacological and lifestyle interventions. CORDELIA includes 196,632 individuals (ages 18–84, 54% female, 96% born in Spain, 20% with higher education, recruited from 1989 to 2020, with follow-up periods ranging from 5 to 30 years), with DNA samples available for 117,342 participants (60%). Of the participants, 24% were current smokers, 43% hypertensive, 11% diabetic, 15% medicated with lipid-lowering drugs, 44% overweight, and 27% obese. If not already available, genotyping is being performed using the Axiom™ Spain Biobank array (~ 750,000 variants, including 115,000 specific and 50,000 rare functional variants from the Spanish population). The cohort also includes incident events (coronary heart disease, stroke, heart failure, peripheral artery disease, hypertension, diabetes); date and cause of death; and harmonized data on risk factors (body mass index, waist circumference, lipid profile, blood pressure, glucose, creatinine), lifestyle (smoking, physical activity, diet, alcohol), and socioeconomic status. 99,019 participants (50%) also provide plasma samples. CORDELIA will significantly contribute to understanding the complex interplay of risk factors contributing to cardiovascular disease and advance the fields of precision medicine and public health in Southern European individuals.

**Supplementary Information:**

The online version contains supplementary material available at 10.1007/s10654-025-01229-6.

## Background

The incidence and mortality rates of atherosclerotic cardiovascular disease (ASCVD) are decreasing worldwide and also in Europe, although the absolute number of cases continue to increase due to the aging of population and it remains the most relevant cause of morbidity and mortality [1]. ASCVD risk can be decreased by a healthy lifestyle (not smoking, a balanced diet, regular physical activity) and the available pharmacological treatments for ASCVD risk factors. However, these approaches are often insufficient, and it is essential to have a good understanding of all environmental, genetic, and socioeconomic factors involved in the development of ASCVD [2, 3]. These factors are locally divergent, which justifies the creation of large population cohorts in specific regions in the world [4–6]. Very few large cohorts are available in Southern European and Mediterranean populations [7]. In Spain, the recently launched IMPaCT Precision Medicine initiative is the only cohort study of its kind, aiming to recruit approximately 200,000 participants by 2028 and complete a 10-year follow-up period by 2038 [8]. Southern European populations are scarcely represented in genome-wide association studies (GWAS) in individuals of European ancestry [9]. In addition, it remains unknown whether genetic variants related to ASCVD can improve the prediction of the disease in this population. European and American studies indicate that around two-thirds of cardiovascular events occur in individuals classified as low or moderate risk by current predictive models [10, 11], who therefore do not qualify for intensive preventive strategies. Further research is needed to determine if these genetic variants could also identify individuals who would benefit most from specific lifestyle changes and pharmacological interventions to prevent ASCVD [12].

## The cordelia study: rationale and aims

In this context, the general aim of the CORDELIA Study (*Collaborative cOhorts Reassembled Data to study mEchanisms and Longterm Incidence of chronic diseAses*) is to investigate a comprehensive range of risk factors for ASCVD, including environmental, socioeconomic, clinical, genetic, and other omics data, using information from 35 existing Spanish cohorts. In particular, CORDELIA aims to conduct the largest GWAS on ASCVD in the Southern European population to date, aiming to identify the genetic variants linked to the occurrence of ASCVD in these individuals. It also aims to create polygenic risk scores for ASCVD adapted to the characteristics of the Spanish population, and to assess whether their inclusion in predictive functions improves their capacity to predict the 10-year incidence of ASCVD. CORDELIA also aims to identify subgroups of the population according to their genetic predisposition to disease that would benefit the most from the available pharmacological treatments and lifestyle modifications. All the previous results will be stratified by sex, highlighting the relevance of sex-specific differences in ASCVD, which can lead to more precise and effective prevention strategies.

## Methods

### Study population

The CORDELIA Study includes 196,632 individuals, aged 18 to 84 years, recruited in Spain from 1989 to 2020, who gave their informed consent in their respective cohorts, and a completed follow-up on cardiovascular events between 5 and 30 years. DNA samples are available in 117,342 of the participants (60%). Serum or plasma samples are available in 99,019 of the participants (50%). The general characteristics of the 35 cohorts included in the CORDELIA Study are available in Table 1.

**Table 1:** Characteristics of the cohorts included in the CORDELIA Study (population aged 18–84 years)

Cohort	Region	Sample size			Recruited	Response rate (%)	Sampling	Sampling procedure	References
		Total	DNA available	Plasma available					
Registre Gironí del Cor (REGICOR)	Catalonia	11,632	10,980	10,980	1995–2018	72	Population-based	Two-stage cluster: age, sex	[13]
Auto-cribado del riesgo cardiovascular (ACRISC)	Catalonia	743	728	728	2015–2017	60	Population-based	Random	[14]
Cohort de dones post-climatèriques de l’àrea de Barcelona (BARCOS)	Catalonia	473	473	473	1989–2010	100	Population-based	Unselected	[15]
Aragón Workers’ Health Study (AWHS)	Aragon	5,670	5,670	5,670	2009–2010	90	Workers	Unselected, consecutive inclusion of volunteers	[16]
Estudio clínico‐epidemiológico salmantino de las patologías del corazón (SALMANTICOR)	Castilla y León	1,993	1,993	1,993	2015–2018	58	Population-based	Three-stage cluster: age, sex, residence	[17]
Prevalence of diabetes and impaired glucose regulation (DI@BET.ES)	Spain	5,419	4,687	-	2007–2010	56	Population-based	Random. Cluster: town	[18]
Study of diet and health in a population from southern Spain (PIZARRA)	Andalusia	2,089	1,422	2,000	2007–2012	75	Population-based	Random	[19]
Evolución de pacientes con PREDiabetes en Atención Primaria de Salud (PREDAPS)	Spain	2,022	341	341	2012	70	Population-based	Unselected, consecutive inclusion of patients	[20]
Dieta y Riesgo de Enfermedad Cardiovascular en España (DRECE)	Spain	3,465	-	-	1991	96	Population-based	Two-stage cluster: age, sex	[21]
Estudio sobre Nutrición y RIesgo CArdiovascular (ENRICA)	Spain	13,018	-	-	2008–2010	51	Population-based	Three-stage cluster: province, municipality,	[22]
European Prospective Investigation into Cancer and Nutrition (EPIC): EPIC-Granada, EPIC-Gipuzkoa, EPIC-Murcia, EPIC-Navarra	Andalusia	7,864	6,448	6,879	1992–1996	88	Population-based	Healthy volunteers	[23]
	Basque Country	8,417	8,300	8,300					
	Murcia	8,515	7,920	8,145					
	Navarra	7,953	7,806	8,031					
Encuesta de Factores de Riesgo Cardiovascular de Murcia (EFRCV)	Murcia	3,089	-	-	1992–1993	61	Population-based	Multi-stage random within clusters: residence, age, sex	[24]
MultiCase-Control Study-Spain (Control-MCC-Spain)	Spain	4,077	2,823	-	2008–2013	53	Population-based	Population-based randomly selected controls, frequency matched to cancer cases by age, sex, and region	[25]
COR SA a les Illes Balears (CORSAIB)	Balearic Islands	1,685	-	-	1999–2000	92	Population-based	Random in clusters: region	[26]
Estació de Monitorització de Malalties Arterioscleròtiques (EMMA)	Catalonia	35,175	-	-	1997–2002	100	Population-based	Random	[27]
PERipheral ARTery disease study (ARTPER)	Catalonia	3,736	1,809	-	2006–2008	100	Population-based	Random in clusters: primary care center	[28]
National Observatory of Atherosclerosis in Nephrology (NEFRONA)	Spain	3,004	3,000	3,000	2009–2012	90	Hospital-based	Cluster: kidney disease stage	[29]
Estudio de intervención longitudinal prospectivo sobre enfermedades vasculares y renales ocultas (ILERVAS)	Catalonia	8,330	7,229	6,103	2015–2018	83	Population-based	Cluster: age, primary care center	[30]
Diabetes y prediabetes en Asturias (ASTURIAS)	Asturias	1,034	232	232	1998–1999	64	Population-based	Two-stage cluster: region, age	[31]
Cardiovascular, Diabetes, Cáncer (CDC de Canarias)	Canary Islands	7,160	7,081	7,036	2000–2005	70	Population-based	Two-stage cluster: island, region	[32]
Harmonizing Equations of Risk in Mediterranean Countries-Extremadura (HERMEX)	Extremadura	2,833	-	-	2007–2009	81	Population-based	Random, stratified by age and sex	[33]
Evaluation of non-traditional risk factors of cardiometabolic and other chronic diseases in a general population from Spain (HORTEGA)	Castilla y León	1,475	1,475	-	1999–2000	62	Population-based	Random, stratified by age and sex	[34]
Riesgo Cardiovascular en Navarra (NAVARRA 93)	Navarra	1,480	-	-	1993	81	Population-based	Three-stage cluster: age, sex, region	[35]
Estudio de Riesgo Vascular en Navarra (RIVANA)	Navarra	4,164	892	4,164	2004–2005	74	Population-based	Random: stratified by age and sex	[36]
Riesgo de Enfermedad Cardiovascular en Castilla y León (RECCYL)	Castilla y León	3,761	3,761	3,761	2004	87	Population-based	Two-stage stratified random sampling (region, rurality)	[37]
PREvalencia de Diabetes Mellitus y Riesgo Cardiovascular (PREDIMERC)	Madrid Autonomous Region	2,268	649	2,000	2007	56	Population-based	Two-stage stratified random sampling (socioeconomic status, primary care center)	[38]
Estudio para la identificación de la población española de riesgo cardiovascular y renal (IBERICAN)	Spain	8,035	6,008	-	2014–2018	87	Population-based	Unselected, consecutive in primary care centers	[39]
A Estrada Glycation and Inflammation Study (AEGIS)	Galicia	1,486	1,315	-	2012–2015	68	Population-based	Random in cluster: age, sex, neighborhood	[40]
Prevalence of diabetes mellitus and impaired glucose metabolism in the adult population of the Basque Country (DI@BETES-Euskadi)	Basque Country	567	567	-	2010–2012	68	Population-based	Random in cluster: primary care center	[41]
CARdiovascular GENetic risk score for Risk Stratification of patients positive for SARS-CoV-2 (COvid 19) virus (CARGENCORS)	Catalonia	2,838	2,575	-	2020	80	Population-based	Consecutive: hospital and primary care COVID-19 triage	[42]
Genomes for Life (GCAT)	Catalonia	19,187	19,183	19,183	2014–2017	NA	Population-based	Unselected, volunteers and blood donors	[43]
RIesgo CARdiovascular y eventos cardiovasculares en la población general del área sanitaria de TOledo (RICARTO)	Castilla La Mancha	1,975	1,975	-	2011–2020	32	Population-based	Random: stratified by age, sex, and region	[44]
**All cohorts**		196,632	117,342	99,019	1989–2020	81			

### Genotype data

Genotype information for five participating cohorts has already been provided. Specifically, AWHS, Control-MCC-Spain, and HORTEGA cohorts have utilized the Infinium™ Global Screening Array-24 BeadChip platform, with their genotypes aligned to the GRCh38/hg38 reference genome and imputed using the TOPMed imputation panel with an r2 quality metric. Additionally, the NEFRONA and CARGENCORS cohorts have employed the Axiom™ Spain Biobank Array-1, with their genotypes referenced to the GRCh37/hg37 genome and imputed using the TOPMed imputation panel with an r3 quality metric.

For the rest of the cohorts, obtaining genotype information is underway. DNA extraction in the CORDELIA study samples was performed using a liquid–liquid method with the FlexiGene DNA Kit reagent from QIAGEN (QIAGEN, Valencia, California, USA). The performance of the extraction, and the quality of the obtained DNA samples were assessed using an Infinite M200 spectrophotometer (Tecan Group Ltd., Männedorf, Switzerland). DNA was quantified with the NanoQuant absorbance method. DNA integrity was also evaluated using 1% agarose gels and quantified by Picogreen (fluorescence) prior to genotyping. During the genotyping process, the samples were quantified with spectrophotometry to verify appropriate amplification. The fragmentation step was monitored with 4% agarose gels. RECCYL was the only cohort that provided isolated DNA samples with verified quality control (performed in the DNA National Bank, University of Salamanca, Salamanca, Spain).

DNA samples are being genotyped at the Fundación Pública Galega de Medicina Xenomica (Santiago de Compostela, Spain), using the Axiom™ Spain Biobank Array-2 (ThermoFisher Scientific, Cornellà de Llobregat, Spain) [45]. This array contains 757,836 variants selected to provide functional genomic coverage, including 114,898 specific variants and 50,536 rare functional variants from the Spanish population. Total genomic DNA (200 ng) is amplified and fragmented to 25–125 base pair oligomers. These fragments are purified and transferred to the GeneTitan Multichannel Instrument for automated hybridization, staining, washing, and scanning. Genotyping calling and clustering is performed with the software Axiom Analysis Suite v5.3.0.45.

Regarding quality control: (1) probes showing genotyping problems (e.g., poor cluster separation) are not included; (2) markers with minor allele frequency < 1%, genotyping rate < 98%, and deviating from Hardy–Weinberg equilibrium (*p*-value < 10^–6^ with a mid-*p* adjustment) are removed [46]; and (3) samples with < 98% genotyping rate and a heterozygosity rate that deviates more than 5 standard deviations from the mean heterozygosity rate of the study are also excluded. A subgroup of > 100,000 independent autosomal SNPs (minor allele frequency > 5% excluding regions with high linkage disequilibrium [47], pruned with a window size of 1000 markers, step size of 80, and r^2^ of 0.1) will be used to detect kinship (by identity-by-descendent estimation, removing individuals at first and second degree of kinship) and population stratification. After removing related individuals and outliers, the first 10 ancestry-informative genetic principal components will be calculated, to be used as covariates in the association analyses. Unrelated individuals will be analyzed in the Admixture software to assess their ancestry using independent markers overlapping the 1000 Genomes Project superpopulations [48, 49]. Individuals will be assigned to a specific ancestry if their probability of being part of that group is ≥ 80%. Otherwise, they will be classified as “mixed” individuals. Finally, after quality control, imputation of genetic variants will be performed using the TOPMed r3 reference panel (GRCh38) [50]. The post-imputation filtering applied will be an INFO score ≥ 0.8 and minor allele frequency > 1%.

### Study variables: harmonization

#### Administrative variables

The components cohorts’ questionnaires were based on standardized surveys by the World Health Organization [51]. The following basic administrative variables are available for all cohorts: center code (a consecutive number starting at 01 for each cohort), cohort name (the acronym recognized and published by the cohort investigators), participant number (pseudonymized identifiers used by the cohorts), date of inclusion (the date each participant signed the informed consent and was first examined), date/year of birth, age at inclusion (self-reported or calculated as the difference between date of birth and inclusion), and sex (female or male phenotype, collected by the cohort personnel). Some cohorts also collected information on country of origin (self-reported), civil status (harmonized to single, married/with a partner, divorced/separated, widow, or not available), and city/town/village of residence.

#### Clinical outcomes

Regarding the clinical outcomes of the study, there is a diagnosis recorded in a medical record or a medical report indicating that the participant has developed the condition plus the diagnosis date. The cardiometabolic events identified in the follow-up of the cohorts participating in the meta-cohort include: fatal and non-fatal acute myocardial infarction, exertional or unstable angina, fatal and non-fatal stroke, heart failure, peripheral artery disease, chronic kidney disease, diabetes and hypertension in initially non-diabetic or non-hypertensive participants. The definition of these non-fatal events in the follow-up is performed by clinical committees of the individual cohorts (following the criteria agreed by the Committee for the Harmonization of Outcomes and Variables of the CORDELIA Study). The following International Classification of Diseases (ICD) codes are being considered: coronary heart disease (ICD-9: 410–414; ICD-10: I20-I25); cerebrovascular disease (ICD-9: 430–437.0; ICD-10: I60-I64); heart failure (ICD-9: 402, 404, 428; ICD-10: I11, I13.0, I13.2, I50), and peripheral artery disease (ICD-9: 440.2; ICD-10: I70.2, I70.3, I70.92, I73.9, E10.5, E11.5). Incident cases of hypertension and type-2 diabetes are also being collected. The CORDELIA infrastructure enables researchers to propose the future collection of data on additional fatal and non-fatal chronic conditions. Fatal events, as well as the primary cause of death for deceased participants, are verified against the official databases of the Spanish National Institute of Statistics’ Mortality Registry and the Spanish hospital discharges dataset (“*Conjunto Mínimo Básico de Datos*”). The most recent vital status (alive, dead, or unknown) of all cohort participants is available.

The previous history of cardiovascular morbidities of the study participants is also available. Following the standard questionnaires set by the World Health Organization, participants were asked whether their doctors had informed them that they suffered from any of the following: heart attack/myocardial infarction, stroke/brain hemorrhage/brain embolism/thrombosis, angina pectoris, arterial hypertension/high blood pressure, hypercholesterolemia/high blood cholesterol levels, or diabetes. They were also queried about any doctor-prescribed drug treatments they were taking to manage blood pressure, diabetes, or cholesterol.

Given that the definitions of hypertension and diabetes have varied since 1989 (the recruitment year in the oldest cohort), we developed a harmonized algorithm for history of hypertension and diabetes at baseline in all the participating cohorts based on the history of morbidities reported by the participants, some clinical examinations (blood pressure, fasting glucose; see following paragraph), and drug use information (see Supplemental Tables 1 and 2, respectively). By doing so, we maximize the number of participants with valid information on their history of hypertension and diabetes as a covariate for subsequent analyses and ensure these outcomes are defined according to current clinical guidelines.

**Table 2:** Population characteristics of the CORDELIA aggregated cohorts

	All participants (*n* = 196,632)	Female (*n* = 105,513)	Male (*n* = 91,119)	Valid data (*n*, %)
Age, years (mean ± SD)	52.5 ± 13.2	52.2 ± 13.3	52.8 ± 13.1	196,632 (100.0)
Female sex (*n*, %)	105,513 (53.7%)	105,513 (100%)	0 (0.00%)	196,632 (100.0)
Born in Spain (*n*, %)	77,291 (95.5%)	42,416 (95.0%)	34,875 (96.1%)	80,825 (41.1)
Civil status:				102,474 (52.1)
Single (*n*, %)	13,653 (13.3%)	7,623 (12.9%)	6,029 (13.8%)	
Married/partnered (*n*, %)	78,133 (76.2%)	43,197 (73.4%)	34,936 (80.1%)	
Divorced/separated (*n*, %)	5,600 (5.46%)	3,728 (6.33%)	1,872 (4.29%)	
Widow (*n*, %)	5,088 (4.97%)	4,326 (7.35%)	762 (1.75%)	
Educational attainment:				131,186 (66.7)
None/functionally literate (*n*, %)	20,788 (15.8%)	13,865 (18.8%)	6,923 (12.1%)	
Elementary/primary school (*n*, %)	47,311 (36.1%)	26,824 (36.3%)	20,487 (35.7%)	
High/secondary school (*n*, %)	37,392 (28.5%)	19,402 (26.3%)	17,990 (31.4%)	
University studies (*n*, %)	25,695 (19.6%)	13,755 (18.6%)	11,940 (20.8%)	
Tobacco use:				193,457 (98.4)
Never smoker (*n*, %)	101,078 (52.2%)	68,902 (66.4%)	32,176 (35.9%)	
Current smoker/ex-smoker for < 1 year (*n*, %)	47,285 (24.4%)	19,588 (18.9%)	27,697 (30.9%)	
Ex-smoker for ≥ 1 year (*n*, %)	45,094 (23.3%)	15,242 (14.7%)	29,852 (33.3%)	
Participants with hypertension (*n*, %)	84,085 (42.8%)	40,347 (38.3%)	43,738 (48.0%)	196,518 (99.9)
Systolic blood pressure (minimum), mmHg (mean ± SD)	129 ± 19.6	126 ± 20.4	132 ± 18.0	164,895 (83.9)
Diastolic blood pressure (minimum), mmHg (mean ± SD)	77.8 ± 10.5	76.1 ± 10.4	79.7 ± 10.3	164,895 (83.9)
Heart rate, beats/min (mean ± SD)	71.1 ± 12.0	72.5 ± 11.9	69.6 ± 12.1	96,342 (49.0)
Participants with diabetes (*n*, %)	21,644 (11.0%)	9,654 (9.18%)	11,990 (13.2%)	196,040 (99.7)
Glucose, mg/dL (mean ± SD)	99.2 ± 27.3	95.9 ± 24.4	103 ± 29.7	138,870 (70.6)
Total cholesterol, mg/dL (mean ± SD)	210 ± 41.3	212 ± 41.2	207 ± 41.2	155,414 (79.0)
HDL cholesterol, mg/dL (mean ± SD)	54.7 ± 14.6	59.6 ± 14.7	49.5 ± 12.6	147,016 (74.8)
Participants medicated with cholesterol-lowering drugs (*n*, %)	21,919 (15.5%)	10,600 (14.4%)	11,319 (16.5%)	141,869 (72.1)
Triglycerides, mg/dL (median [1 st-3rd quartile])	98.3 [72.0–139]	89.5 [66.0–124]	110 [80.0–155]	148,351 (75.4)
Body mass index, kg/m^2^ (median [1 st-3rd quartile])	27.3 [24.6–30.4]	26.9 [23.8–30.5]	27.7 [25.3–30.2]	193,836 (98.6)
Categories for body mass index:				193,836 (98.6)
< 18.5 kg/m^2^ (*n*, %)	1,093 (0.56%)	831 (0.80%)	262 (0.29%)	
18.5–24.9 kg/m^2^ (*n*, %)	54,794 (28.3%)	35,179 (33.9%)	19,615 (21.8%)	
25.0–29.9 kg/m^2^ (*n*, %)	84,920 (43.8%)	38,686 (37.3%)	46,234 (51.4%)	
30.0–39.9 kg/m^2^ (*n*, %)	50,268 (25.9%)	27,075 (26.1%)	23,193 (25.8%)	
≥ 40.0 kg/m^2^ (*n*, %)	2,761 (1.42%)	2,044 (1.97%)	717 (0.80%)	
Waist circumference, cm (mean ± SD)	96.1 ± 23.5	91.5 ± 22.5	102 ± 23.5	140,541 (71.5)
Participants with abdominal obesity (*n*, %)	76,433 (54.4%)	36,413 (48.0%)	40,020 (61.9%)	140,541 (71.5)
Creatinine, mg/dL (mean ± SD)	0.87 ± 0.30	0.76 ± 0.25	0.98 ± 0.32	91,282 (46.4)
Glomerular filtration rate, mL/min/1.73 m^2^ (mean ± SD)	90.8 ± 18.2	91.3 ± 18.3	90.3 ± 18.1	91,282 (46.4)

#### Clinical measurements and laboratory determinations

The clinical examinations at the baseline visit include measurements of weight (kg), height (cm), body mass index (BMI, kg/m^2^), waist circumference (cm), heart rate (beats/minute, a single measurement or the average of 2–3 measurements), systolic blood pressure (SBP, mmHg; one or two readings, retaining the lowest), and diastolic blood pressure (DBP, mmHg; one or two readings, retaining the lowest). Participants were categorized according to their BMI values as underweight (< 18.5 kg/m^2^), normal weight (18.5 to < 25.0 kg/m^2^), overweight (25.0 to < 30.0 kg/m^2^), obese (30.0 to < 40.0 kg/m^2^), and morbidly obese (≥ 40 kg/m^2^). Abdominal obesity was defined as a waist circumference of ≥ 89 cm (female) and ≥ 103 cm (male).

Laboratory variables were measured in blood samples taken after an overnight fast, performed in reference laboratories on fresh plasma/serum aliquots stored at −80ºC until use, in samples not previously thawed. Triglycerides, glucose, total cholesterol, and high-density lipoprotein (HDL) cholesterol were measured using enzymatic methods, and creatinine using the Jaffe method. All local laboratories met external quality control standards [13, 26, 28, 32, 33, 36, 52–55]. Lipid profile and glucose measurements in 8 of the cohorts (ARTPER, CDC, CORSAIB, HERMEX, PREDIMERC, RECCYL, REGICOR, RIVANA) were validated for concordance with a central laboratory [56]. The values for triglycerides, glucose, creatinine, total cholesterol, and HDL cholesterol are expressed in mg/dL. Low-density lipoprotein (LDL) cholesterol was calculated using the Friedewald formula (LDL cholesterol = total cholesterol – HDL cholesterol – triglycerides/5) when triglycerides were < 300 mg/dL or otherwise directly determined. When lipid profile and glucose values were originally in mmol/L, they were converted to mg/dL by multiplying by 88.57 for triglycerides, 18.016 for glucose, and 38.67 for total, HDL, and LDL cholesterol. When creatinine was provided in µmol/L, it was converted to mg/dL by multiplying by 0.0113. The estimated glomerular filtration rate for participants with creatinine values (in mL/min/1.73 m^2^) was calculated using the Chronic Kidney Disease Epidemiology Collaboration formula [57]. After harmonization, we defined the following valid data ranges for continuous cardiovascular risk factors: 15–50 kg/m^2^ for BMI, 60–150 cm for waist circumference, 40–250 mmHg for SBP, 20–130 mmHg for DBP, 30–1000 mg/dL for triglycerides, 30–350 mg/dL for glucose, 100–450 mg/dL for total cholesterol, 20–130 mg/dL for HDL cholesterol, and 0.2–5.0 mg/dL for creatinine.

#### Socioeconomic and lifestyle variables

Data on these variables was available from the baseline visit of each cohort. Information on educational attainment has been harmonized into the following categories: none/functionally literate (illiterate, no studies, or only can read and write), elementary/primary school (compulsory basic education), high/secondary school (including “*Bachillerato Unificado Polivalente*”, “*Curso de Orientación Universitaria*”, vocational training, “*Bachillerato*”, and university access courses), university studies, and not available. Age at completion of last studies is also available for some cohorts. Smoking status has been harmonized as follows: current smoker/ex-smoker of less than one year (anyone who smoked ≥ 1 cigarette/day was considered a smoker), ex-smoker of one year or more, never smoker, not available. Additionally, some cohorts provided details on the number of cigarettes per day participants currently smoke or smoked in the past (for smokers and ex-smokers), the age at which participants began smoking (for smokers and ex-smokers), and the age at which ex-smokers quitted smoking. Leisure-time physical activity estimates for the participants were harmonized to average kcal/day whenever possible. When data were reported in metabolic equivalents of task per minute per day, these values were converted to kcal/day by multiplying those values by the participant’s weight in kilograms. Dietary data, whenever possible, were used to construct a Mediterranean diet adherence score, ranging from 1 to 14, with higher scores indicating greater adherence. The diet score positively rated the intake of virgin olive oil, fruits, vegetables, “sofrito” (sauce or base made from sautéed vegetables such as tomatoes, onions, garlic, and peppers), legumes, mixed nuts, fish, poultry, and moderate doses of wine; and it negatively rated the intake of red and processed meats, butter/cream, sugar-sweetened beverages, and non-homemade sweets/pastries [58].

Additional details on the collected variables (whether tobacco use, weight, height, physical activity, and diet information was collected by trained personnel or self-reported; technical details regarding biological samples; and the detailed methodology and equipment used to measure blood pressure and laboratory determinations) is available in Supplemental Table 3.

**Table 3:** Characteristics of the participants of individual cohorts in CORDELIA

	Sample size	Age, years (mean ± SD)	Female (%)	Born in Spain (%)	%Univ. Education (%)	Current smokers (%)	HT (%)	SBP, mmHg (mean ± SD)	DBP, mmHg (mean ± SD)	HR, beat/m (mean ± SD)	Diabetes (%)	Glucose, mg/dL (mean ± SD)	TC, mg/dL (mean ± SD)	HDL-C, mg/dL (mean ± SD)	LDL-C, mg/dL (mean ± SD)	Trig., mg/dL median [1 st-3rd quartile]	Chol.-Lowering treat. (%)	BMI, kg/m^2^ (mean ± SD)	Creat. mg/dL (mean ± SD)
REGICOR	11,632	54 ± 13	52	97	18	24	42	127 ± 20	78 ± 11	69 ± 11	14	101 ± 25	215 ± 42	52 ± 14	141 ± 38	93 [69–129]	10	27 ± 5	0.9 ± 0.2
ACRISC	743	48 ± 10	51	-	44	24	22	109 ± 17	73 ± 11	68 ± 11	6	93 ± 19	197 ± 34	57 ± 15	118 ± 30	96 [71–131]	8	26 ± 4	-
BARCOS	473	58 ± 8	100	76	-	14	20	129 ± 17	77 ± 10	122 ± 35	4	97 ± 17	223 ± 38	63 ± 16	138 ± 36	91 [68–116]	3	26 ± 4	0.8 ± 0.2
AWHS	5,670	47 ± 9	7	-	-	37	34	125 ± 15	83 ± 10	71 ± 12	5	94 ± 19	208 ± 37	52 ± 11	128 ± 31	120 [85–178]	8	27 ± 4	1.0 ± 0.1
SALMANTICOR	1,993	55 ± 16	54	96	24	20	60	140 ± 19	87 ± 11	68 ± 11	12	95 ± 27	183 ± 33	54 ± 14	106 ± 29	97 [71–138]	25	26 ± 4	0.8 ± 0.2
DI@BET.ES	5,419	50 ± 17	58	91	16	26	44	129 ± 20	75 ± 11	-	12	98 ± 26	197 ± 39	52 ± 13	105 ± 30	102 [74–142]	12	28 ± 5	0.8 ± 0.2
PIZARRA	2,089	51 ± 10	61	99	7	26	45	133 ± 21	78 ± 11	72 ± 11	14	103 ± 24	207 ± 40	55 ± 15	128 ± 37	103 [74–153]	12	29 ± 5	0.8 ± 0.2
PREDAPS	2,022	58 ± 10	51	95	17	17	81	130 ± 16	79 ± 10	73 ± 10	0	98 ± 13	210 ± 37	56 ± 15	129 ± 33	110 [80–149]	100	29 ± 5	0.8 ± 0.2
DRECE	3,465	37 ± 12	52	-	12	43	22	120 ± 17	75 ± 12	-	6	95 ± 22	201 ± 44	54 ± 14	124 ± 38	94 [69–138]	5	26 ± 4	1.0 ± 0.2
ENRICA	13,018	47 ± 17	53	94	29	27	36	127 ± 18	74 ± 11	70 ± 11	10	93 ± 20	197 ± 38	53 ± 14	122 ± 32	93 [67–131]	44	27 ± 5	0.9 ± 0.2
EPIC-Granada	7,864	50 ± 9	77	-	12	19	26	125 ± 19	78 ± 11	77 ± 12	8	82 ± 44	231 ± 41	54 ± 14	145 ± 35	142 [97–213]	-	29 ± 5	0.8 ± 0.2
EPIC-Gipuzkoa	8,417	50 ± 8	51	-	9	30	20	125 ± 19	77 ± 11	69 ± 11	6	81 ± 9	231 ± 43	56 ± 15	152 ± 38	97 [65–142]	-	27 ± 4	0.8 ± 0.2
EPIC-Murcia	8,515	49 ± 8	68	-	15	25	26	124 ± 17	77 ± 10	74 ± 11	6	89 ± 19	222 ± 41	54 ± 14	147 ± 37	89 [62–124]	-	28 ± 4	0.7 ± 0.2
EFRCV	3,089	40 ± 13	51	-	10	41	35	126 ± 18	76 ± 12	-	8	-	193 ± 45	62 ± 17	-	96 [70–141]	5	27 ± 4	-
Control-MCC-SPAIN	4,077	63 ± 12	50	95	21	19	39	130 ± 18	75 ± 10	-	16	89 ± 33	205 ± 36	58 ± 13	85 ± 20	118 [87–164]	25	27 ± 4	0.8 ± 0.2
CORSAIB	1,685	54 ± 11	52	-	8	30	45	130 ± 20	80 ± 10	75 ± 11	12	104 ± 30	217 ± 41	53 ± 13	139 ± 36	107 [77–153]	6	28 ± 5	-
EMMA	35,175	58 ± 12	52	-	-	19	67	137 ± 21	79 ± 9	-	19	104 ± 35	224 ± 42	53 ± 14	149 ± 40	96 [72–132]	9	28 ± 4	-
ARTPER	3,736	64 ± 9	54	97	7	17	65	136 ± 18	78 ± 10	73 ± 11	20	107 ± 29	216 ± 39	55 ± 14	135 ± 34	109 [81–151]	31	29 ± 5	-
NEFRONA	3,004	57 ± 13	40	-	-	20	88	141 ± 22	81 ± 12	-	26	106 ± 35	184 ± 39	50 ± 15	107 ± 35	105 [82–128]	57	28 ± 5	1.9 ± 1.0
ILERVAS	8,330	57 ± 6	51	-	-	30	56	131 ± 17	82 ± 10	-	0	-	206 ± 38	57 ± 15	145 ± 26	137 [103–189]	18	29 ± 5	0.8 ± 0.2
ASTURIAS	1,034	53 ± 13	54	-	-	-	43	134 ± 22	84 ± 13	-	13	100 ± 24	229 ± 41	56 ± 14	150 ± 37	99 [71–141]	-	28 ± 5	1.0 ± 0.2
CDC Canarias	7,160	43 ± 13	56	98	14	26	32	119 ± 19	75 ± 12	73 ± 11	10	97 ± 25	203 ± 41	51 ± 13	127 ± 37	99 [71–146]	39	27 ± 5	-
HERMEX	2,833	51 ± 15	54	98	12	32	38	124 ± 23	72 ± 11	72 ± 11	14	104 ± 25	208 ± 38	56 ± 14	129 ± 33	93 [68–132]	17	29 ± 5	0.8 ± 0.2
HORTEGA	1,475	52 ± 19	50	-	28	23	40	131 ± 21	79 ± 11	-	4	-	201 ± 38	56 ± 19	109 ± 39	150 [106–212]	7	26 ± 4	0.9 ± 0.2
NAVARRA 93	1,480	40 ± 14	50	99	15	47	24	118 ± 18	76 ± 11	69 ± 10	3	96 ± 17	213 ± 43	58 ± 16	133 ± 40	91 [67–125]	23	26 ± 4	-
EPIC-Navarra	7,953	50 ± 8	52	-	9	33	20	-	-	-	3	77 ± 17	228 ± 42	54 ± 14	153 ± 37	97 [71–133]	-	28 ± 4	0.8 ± 0.2
RIVANA	4,164	54 ± 13	55	96	22	27	40	128 ± 19	78 ± 10	52 ± 14	12	102 ± 22	212 ± 37	64 ± 16	126 ± 34	92 [69–132]	12	27 ± 4	0.9 ± 0.2
RECCyL	3,761	52 ± 18	52	98	-	24	41	128 ± 19	76 ± 11	-	10	98 ± 26	209 ± 41	59 ± 15	131 ± 36	88 [64–126]	11	27 ± 5	0.9 ± 0.2
PREDIMERC	2,268	50 ± 13	52	87	24	26	38	127 ± 19	76 ± 10	71 ± 11	9	99 ± 22	201 ± 36	57 ± 15	123 ± 32	90 [65–128]	12	27 ± 5	0.8 ± 0.2
IBERICAN	8,035	58 ± 15	55	-	14	19	56	129 ± 16	77 ± 10	73 ± 11	21	102 ± 28	195 ± 39	55 ± 15	118 ± 35	107 [78–151]	37	28 ± 5	0.8 ± 0.3
AEGIS	1,486	52 ± 17	55	94	12	21	41	128 ± 16	76 ± 9	65 ± 11	12	94 ± 23	197 ± 38	59 ± 17	115 ± 32	95 [69–132]	19	28 ± 5	0.7 ± 0.2
DI@BET.ES-EUSKADI	567	52 ± 15	53	95	24	24	42	132 ± 21	80 ± 12	-	10	92 ± 22	206 ± 39	61 ± 17	124 ± 35	91 [66–125]	11	26 ± 5	0.8 ± 0.2
CARGENCORS	2,838	57 ± 13	55	-	-	9	45	129 ± 17	77 ± 11	-	15	101 ± 29	199 ± 41	56 ± 16	122 ± 35	108 [76–153]	-	28 ± 5	0.8 ± 0.3
GCAT	19,187	51 ± 7	59	96	38	22	27	122 ± 15	77 ± 10	74 ± 11	4	92 ± 18	209 ± 36	59 ± 15	130 ± 32	92 [68–130]	7	27 ± 5	0.8 ± 0.2
RICARTO	1,975	50 ± 16	56	-	22	23	33	123 ± 17	72 ± 11	74 ± 11	9	89 ± 19	193 ± 36	57 ± 15	114 ± 33	92 [69–130]	20	27 ± 5	0.8 ± 0.2
All	196,632	52 ± 13	54	96	20	24	43	129 ± 20	78 ± 11	71 ± 12	11	99 ± 27	210 ± 41	55 ± 15	132 ± 38	98 [72–139]	15	28 ± 5	0.9 ± 0.3

*BMI* body mass index, *Chol.-lowering treat.* cholesterol-lowering treatment, *Creat*. Creatinine, *DBP* diastolic blood pressure, *HChol* hypercholesterolemia, *HDL-C* HDL cholesterol, *HR* heart rate, *HT* hypertension, *LDL-C* LDL cholesterol, *SBP* systolic blood pressure, *TC* total cholesterol, *Trig*. Triglycerides, *Univ*. university

All cohorts’ data have been harmonized and integrated into a single, curated database hosted at the Hospital del Mar Research Institute in Barcelona. Whenever between-cohort heterogeneity is substantial, phenotype data analyses will be conducted in the pooled CORDELIA database using mixed-effects models (considering the cohort as a random effect variable), and genotype analyses will employ a meta-analysis framework. According to the consortium’s by-laws, CORDELIA investigators will access the integrated dataset until the end of 2026. Thereafter, any researcher may request access by submitting a standardized application form available at www.cordeliaproject.net; each request will undergo scientific and ethical review to ensure proper handling of participant data.

### GWAS sample size

Accepting an alpha risk of 5 × 10^–8^ (the standard genome-wide significance threshold), a statistical power of 80% in a two-sided test, and a 10-year incidence rate of coronary events of 3.7% in the group of participants not exposed to the risk allele, 117,342 individuals allow the identification of the following relative risks depending on the risk allele frequency (AF): ≥ 1.84 (AF 1%), ≥ 1.38 (AF 5%), ≥ 1.28 (AF 10%), ≥ 1.24 (AF 15%), ≥ 1.21 (AF 20%), ≥ 1.20 (AF 25%), ≥ 1.19 (AF 30%), ≥ 1.18 (AF 40%), and ≥ 1.17 (AF 50%). In the sex-stratified analyses (54% of the participants are female), under the same assumptions, our population allows the identification of the following relative risks: ≥ 2.15 (AF 1%), ≥ 1.52 (AF 5%), ≥ 1.38 (AF 10%), ≥ 1.32 (AF 15%), ≥ 1.29 (AF 20%), ≥ 1.27 (AF 25%), ≥ 1.26 (AF 30%), ≥ 1.24 (AF 40%), and ≥ 1.24 (AF 50%).

## Results

### Characteristics of the study population

As described in Table 2, participants in the CORDELIA study were, on average, 52 years old, with female participants comprising 54% of the group. According to cohorts that provided this information, the majority were born in Spain (96%). 20% had attained a university degree. Smoking status showed that 52% had never smoked, and 24% were current smokers. Cardiovascular and metabolic conditions were prevalent, with 43% having hypertension, 11% diagnosed with diabetes, and 15% taking cholesterol-lowering drugs. The average triglyceride level was 98 mg/dL. The percentages of participants who were overweight, obese, and morbidly obese were 44%, 26%, and 1.4%, respectively. Abdominal obesity was present in 54% of the participants. The mean participants’ estimated glomerular filtration rate was 90.8 mL/min/1.73 m^2^. A description of the variables in each of the CORDELIA cohorts is available in Table 3. The proportion of participants with valid data for the study variables in each of the individual cohorts is described in Supplemental Table 4.

## Discussion

### Findings to date

This cohort profile is the first joint publication of the CORDELIA Study. However, as evidenced by several high-impact publications, its participating cohorts have substantially contributed to the epidemiological understanding of various health outcomes, including acute myocardial infarction [59, 60], stroke [61], peripheral artery disease [62], subclinical atherosclerosis [63], cancer [64–67], neurodegenerative disease [68, 69], diabetes [18], obesity [70, 71], disability [72], sleep-related diseases [73], and COVID-19 [42, 74].

### Strengths and limitations

The CORDELIA Study has several strengths. It is, to date, the largest meta-cohort on cardiovascular disease of South European population with almost 200,000 participants, maximizing the statistical power of the individual cohorts. To our knowledge, it is also one of the largest meta-cohorts combining a comprehensive set of clinical, socioeconomic, and lifestyle variables, DNA samples (available in 117,342 participants, 60%) and serum/plasma samples (available in 99,019 participants, 50%) in European population. CORDELIA represents an exhaustively defined population, aiming to be representative of the Spanish adult population thus improving the generalizability of our findings to Southern European adult populations (genetic ancestry, lifestyle, and clinical variables are expected to be comparable across cohorts). It will also conduct the largest GWAS on ASCVD in Southern European population. Due to its unique characteristics, it can assess the association of certain rare variants that are specific to Spanish or South European population with the occurrence of ASCVD. It will also investigate whether a polygenic risk score can improve the current predictive functions for the 10-year incidence of ASCVD in this same population. Genetic characteristics vary according to ethnicity and location; therefore, it is plausible that calculating a polygenic risk score that considers “local” genetic variants improves risk prediction. Additionally, it will explore whether there are relevant interactions between genetic and lifestyle information on the development of ASCVD that are specific to Southern European population. It is plausible that some interactions are only evident if both the local genetic background and a local environmental exposure are considered together (e.g., Southern European populations have greater adherence to a Mediterranean diet than other regions of the world).

However, it also has limitations. First, inter-cohort data may be heterogeneous, as the cohorts may have used different methods for recruitment, phenotype definition and data collection, which was also performed at distinct times (between 1989 and 2020). To minimize this bias, the Data Management team, in collaboration with the Committee for the Harmonization of Outcomes and Variables of the CORDELIA Study, has performed exhaustive harmonization of the available data (to the lowest common denominator for categorical variables) and the statistical analyses will undergo central validation and be adapted to the nature of the data (whenever possible, mixed models that consider the cohort as a random effect will be used). Regarding genotype heterogeneity and population stratification, these issues will be minimized by analyzing participants’ relatedness and adjusting for genetic principal components. If significant genotype heterogeneity among cohorts is detected, individual GWAS analyses in each participating cohort may be performed and subsequently meta-analyzed [75]. Second, while imputation allows obtaining results for a very high number of genetic variants (about 9–10 million), the number of directly genotyped variants will be about 750,000. To minimize potential biases, imputation will be performed using the comprehensive TOPMed r3 (GRCh38) reference panel and a stringent post-imputation filtering (INFO score ≥ 0.8 and a minor allele frequency > 1%). Third, our data may not be fully representative of the Spanish population due to different designs for population selection (two/three-stage clusters, random, and non-random selections of population-based, hospital-based, and worker studies), the characteristics of the participants in the individual cohorts (some focused on healthy individuals, while others included populations with various pathologies; additionally, each cohort recruited individuals of diverse age ranges) and a recruitment over a period of almost 30 years. The large number of participants included in the meta-cohort may help mitigate these differences, but an exhaustive description of the study population must be provided in each publication derived from our data. Fourth, the follow-up times and the diverse age ranges in the participating cohorts may decrease the capacity of cohorts with shorter follow-ups or younger participants to detect outcomes. To minimize this limitation, all cohorts will conduct a follow-up update of fatal and non-fatal events during 2024, allowing for more homogeneous tracking of the participants across studies. Fifth, anthropometric, lifestyle, and socioeconomic variables in certain cohorts are self-reported, which may increase measurement error. Sixth, although the sample size in such a study in Southern European population is the largest to date, the statistical power to detect associations between rare genetic variants (those with an AF of ≤ 1%) and the outcomes of interest may be limited. Seventh, since CORDELIA leverages existing data and biological samples from 35 cohorts, genotype data will be available for at most 60% of participants, and biological samples will be available for 50%. Biological samples cannot be obtained from cohorts that do not already have stored DNA or serum/plasma samples. Eighth, the definitions of hypertension and diabetes across the cohorts may vary, as they might be based on self-reported information, individual-level measurements, and treatment information. This variation could introduce heterogeneity and bias in assessing these conditions across cohorts. To minimize this limitation, we have developed a harmonized algorithm to maximize the number of participants with available information on their history of hypertension and diabetes (available in 99.9% and 99.7% of the participants, respectively). Finally, while our conclusions could be generalized to Southern European adults with similar characteristics, they may not be extended to populations of other genetic ancestry or different regions.

## Conclusions

The CORDELIA Study marks a significant advancement as the largest cohort for the study of cardiovascular disease within South European adult population to date. CORDELIA also establishes the foundation for the most extensive GWAS on ASCVD in Southern Europe using a well-characterized population with long follow-up. This infrastructure will also support future projects targeting other diseases. CORDELIA aims to enhance the representation of Southern Europeans in genomic studies and to develop tailored polygenic risk scores that refine ASCVD prediction and potentially improve targeted prevention strategies. By integrating comprehensive clinical, environmental, genetic, and socioeconomic data, CORDELIA exemplifies how large-scale collaborative research can advance precision medicine.

## Supplementary Information

Below is the link to the electronic supplementary material.

Supplementary file1 (DOCX 72 KB)

## Abbreviations

ASCVD: Atherosclerotic cardiovascular disease
ACRISC: *Auto-cribado del riesgo cardiovascular*
AEGIS: A Estrada Glycation and Inflammation Study
ARTPER: PERipheral ARTery disease study
ASTURIAS: *Diabetes y prediabetes en Asturias*
AWHS: Aragón Workers’ Health Study
BARCOS: *Cohort de dones post-climatèriques de l’àrea de Barcelona*
CARGENCORS: *CARdiovascular GENetic risk score for Risk Stratification of patients positive for SARS-CoV-2 (COvid 19) virus*
CDC de Canarias: *Cardiovascular, Diabetes, Cáncer*
Control-MCC-Spain: MultiCase-Control Study-Spain (Control participants)
CORSAIB: *COR SA a les Illes Balears*
DIBETES-EUSKADI: Prevalence of diabetes mellitus and impaired glucose metabolism in the adult population of the Basque Country
DRECE: *Dieta y Riesgo de Enfermedad Cardiovascular en España*
EFRCV: *Encuesta de Factores de Riesgo Cardiovascular de Murcia*
EMMA: *Estació de Monitorització de Malalties Arterioscleròtiques*
ENRICA: *Estudio sobre Nutrición y RIesgo CArdiovascular en España*
EPIC: European Prospective Investigation into Cancer and Nutrition
GCAT: Genomes for Life
HERMEX: Harmonizing Equations of Risk in Mediterranean Countries-Extremadura
HORTEGA: Evaluation of non-traditional risk factors of cardiometabolic and other chronic diseases in a general population from Spain
IBERICAN: *Estudio para la identificación de la población española de riesgo cardiovascular y renal*
ILERVAS: *Estudio de intervención longitudinal prospectivo sobre enfermedades vasculares y renales ocultas*
NAVARRA 93: *Riesgo Cardiovascular en Navarra*
NEFRONA: National Observatory of Atherosclerosis in Nephrology
PIZARRA: Study of diet and health in a population from southern Spain
PREDAPS: *Evolución de pacientes con PREDiabetes en Atención Primaria de Salud*
PREDIMERC: *PREvalencia de Diabetes Mellitus y Riesgo Cardiovascular*
RECCYL: *Riesgo de Enfermedad Cardiovascular en Castilla y León*
REGICOR: *Registre Gironí del Cor*
RICARTO: *RIesgo CARdiovascular y eventos cardiovasculares en la población general del área sanitaria de TOledo*
RIVANA: *Estudio de Riesgo Vascular en Navarra*
SALMANTICOR: *Estudio clínico‐epidemiológico salmantino de las patologías del corazón*
