# Supplementary material for: Cohort profile: the CORDELIA study (Collaborative cOhorts Reassembled Data to study mEchanisms and Longterm Incidence of chronic diseAses)
Source: Eur J Epidemiol. 2025 May 12;40(5):581–99. doi: 10.1007/s10654-025-01229-6 (PMC12170779; doi:10.1007/s10654-025-01229-6)
Supplement: Supplementary file 1 — Supplementary file1 (DOCX 72 KB) [file 10654_2025_1229_MOESM1_ESM.docx]

**SUPPLEMENTAL MATERIALS**

**SUPPLEMENTAL TABLES**

**Supplemental Table 1.** Harmonized diagnostic algorithm for hypertension in CORDELIA.

| **Reported antecedents of hypertension** | **Systolic blood pressure** | **Diastolic blood pressure** | **Treatment for hypertension** | **Harmonized criterion for hypertension in CORDELIA** |
| --- | --- | --- | --- | --- |
| Yes | Any answer | Any answer | Any answer | YES |
| No/Missing | ≥140 mmHg | Any answer | Any answer | YES |
| No/Missing | Any answer | ≥90 mmHg | Any answer | YES |
| No/Missing | Any answer | Any answer | Yes | YES |
| No/Missing | <140 mmHg | <90 mmHg | No/Missing | NO |
| No | Missing | Missing | No/Missing | NO |
| Missing | Missing | Missing | No/Missing | MISSING |

**Supplemental Table 2.** Harmonized diagnostic algorithm for diabetes in CORDELIA.

| **Reported antecedents of diabetes** | **Fasting glucose** | **Treatment**  **(oral glucose-lowering drugs or insulin)** | **Harmonized criterion for diabetes in CORDELIA** |
| --- | --- | --- | --- |
| Yes | Any answer | Any answer | YES |
| No/Missing | ≥126 mg/dL | Any answer | YES |
| No/Missing | Any answer | Yes | YES |
| No/Missing | <126 mg/dL / Missing | No/Missing | NO (one or two negative criteria) |
| Missing | Missing | Missing | MISSING |

**Supplemental Table 3.** Additional details on harmonized variables.

| **Cohort** | **Weight and height** | **Biological**  **samples** | **Laboratory tests** | **Blood pressure** | **Tobacco** | **Physical activity** | **Diet data** |
| --- | --- | --- | --- | --- | --- | --- | --- |
| REGICOR | Trained personnel, using calibrated scales and stadiometers | Fasting state | Total cholesterol and triglycerides were measured in serum using enzymatic methods (Roche diagnostics).  HDL-C was measured after ApoB precipitation (Boehringer).  Quality control: Coefficient variation < 5% | In recruitment: using a mercury sphygmomanometer.  Follow up: using an automatic sphygmomanometer.  After 5 minutes of rest.  Average of 2 measurements (3 if one of the values is highly discordant) | Trained personnel | Trained personnel, using the Minnesota questionnaire | Self-reported |
| ACRISC | Trained personnel, using calibrated OMRON scales | Fasting state | Measurements in serum, reagents by ABX-HORIBA | Two measurements separated by two minutes, using an OMRON tensimeter (Omron, Barcelona, Spain) | Self-reported | Self-reported, using the Minnesota questionnaire | Self-reported |
| BARCOS | Trained personnel, using calibrated scales | Fasting state | Measurements performed in the Reference Laboratory of Catalonia | No details available | Trained personnel | Not available | Not available |
| AWHS | Trained personnel. Calibrated scale SECA 778.  Waist circ. was measured using GULICK II 67019 metric tapes, halfway between the iliac crest and the lower ribs. | Fasting state | Glucose, triglyceride, total and HDL cholesterol: enzymatic analysis in ILAB 650 analyzer. | Average of 3 consecutive automatic readings, after a 5-min rest period, with an OMRON M10-IT digital blood pressure monitor (OMRON Healthcare Co. Ltd., Japan). | Trained personnel | Trained personnel, using the SUN Study questionnaire | Trained personnel |
| SALMANTICOR | Trained personnel, using calibrated scales | Fasting state | No details available | Blood pressure was directly measured by oscillometry and pulse wave analysis by a VaSera VS-1500® device (Fukuda Denshi) | Self-reported | Self-reported | Self-reported |
| DI@BET.ES | Trained personnel, using SOEHNLE calibrated scales. Waist circ. was measured at the level of the navel. | Fasting state | Glucose: hexokinase enzymatic method. Total cholesterol: cholesterol oxidase enzymatic method. HDL cholesterol: direct method. Triglycerides: glycerol phosphate oxidase enzymatic method. Analyses performed in an Architect C8000 Analyzer (Abbott Laboratories SA, Madrid, Spain). | Average of 2 measurements separated by 1-2 minutes, after several minutes in a seated position, with a calibrated tensimeter Hem-703 C (Omron, Barcelona, Spain).  . | Trained nurses | Trained nurses, using the IPAQ questionnaire | Trained nurse |
| PIZARRA | Trained personnel, using SOEHNLE calibrated scales. Waist circ. was measured at the level of the navel. | Fasting state | Glucose: hexokinase enzymatic method. Total cholesterol: cholesterol oxidase enzymatic method. HDL cholesterol: direct method. Triglycerides: glycerol phosphate oxidase enzymatic method. | Average of 2 measurements separated by 1-2 minutes, after several minutes in a seated position, with a calibrated Omron tensimeter (Omron, Barcelona, Spain). | Trained medical doctors and nurses | Trained medical doctors and nurses, using a non-validated questionnaire | Trained medical doctors and nurses |
| PREDAPS | Trained personnel, using calibrated scales. | Fasting state | Glucose, total cholesterol, HDL-cholesterol, triglycerides, and creatinine were measured by enzymatic colorimetric tests. | Measurement after 3 minutes in resting position. High inter-center variability is expected | Trained personnel | Trained personnel | Trained personnel |
| DRECE | Trained personnel, using calibrated scales. | Fasting state | Total cholesterol: cholesterol oxidase CHOD-PAP14 enzymatic method. HDL cholesterol: direct method, precipitation with phosphotungstic acid and magnesium. Triglycerides: glycerol phosphate oxidase GPO-PAP15 enzymatic method. All these methods were adapted to an autoanalyzer (Hitachi 704, Boehringer Mannheim, Germany). Coefficients of variation: <3%. | Average of 2 measurements in the beginning and at the end of the visit, using a standard mercury sphygmomanometer. | Trained personnel | Trained personnel (number of days/week of moderate and intense practice of sport) | Trained personnel |
| ENRICA | Trained personnel. Measurements were performed using electronic scales (model Seca 841, precision to 0.1 kg), portable extendable stadiometers (model Ka We 44 444Seca), and flexible,  inelastic belt-type tapes | Fasting state | Glucose: glucose oxidase method. Total cholesterol: cholesterol esterase and cholesterol oxidase enzymatic methods. HDL cholesterol: direct method using elimination/catalase. Triglycerides: glycerol phosphate oxidase method. All assays were conducted using the ADVIA 2400 Chemistry System (Siemens). | Average of 2 sets of 3 readings. Each set was performed before and after the interview. Measurements in each set were separated by 1-2 minutes, after 3-5 minutes in a seated position or lying up, using a calibrated Omron M6 tensimeter (Omron, Barcelona, Spain). | Self-reported | Self-reported, using the EPIC-Spain and Nurses’ Health Study | Trained personnel (validated electronic diet history) |
| EPIC-Granada | Trained personnel. Weight: calibrated SC-3301 TEFAL electronic scale. Height: barefoot, Añó-Sayol S1B calibrated stadiometer. Waist circumference: as the narrowest circumference of the torso (natural waist) and at the midpoint (halfway between the iliac crest and the lower ribs) | Fasting (9.5%),  non-fasting (90.5%) | Glucose, creatinine, triglyceride, total and HDL cholesterol were measured by enzymatic colorimetric tests. | Average of 2 readings, using an automatic BOSO oscillometer. | Self-reported | Self-reported, using the validated EPIC-PAQ questionnaire (leisure-time, at work, and at home) | Trained personnel |
| EPIC-Gipuzkoa | Trained personnel. Weight: calibrated SC-3301 TEFAL electronic scale. Height: barefoot, Añó-Sayol S1B calibrated stadiometer. Waist circumference: as the narrowest circumference of the torso (natural waist) and at the midpoint (halfway between the iliac crest and the lower ribs) | Fasting and non-fasting | Glucose, creatinine, triglyceride, total and HDL cholesterol were measured by enzymatic colorimetric tests. | Average of 2 readings, using an automatic BOSO oscillometer. | Self-reported | Self-reported, using the validated EPIC-PAQ questionnaire (leisure-time, at work, and at home) | Trained personnel |
| EPIC-Murcia | Trained personnel. Weight: calibrated SC-3301 TEFAL electronic scale. Height: barefoot, Añó-Sayol S1B calibrated stadiometer. Waist circumference: as the narrowest circumference of the torso (natural waist) and at the midpoint (halfway between the iliac crest and the lower ribs) | Fasting and non-fasting | Glucose, creatinine, triglyceride, total and HDL cholesterol were measured by enzymatic colorimetric tests. | Average of 2 readings, using an automatic BOSO oscillometer. | Self-reported | Self-reported, using the validated EPIC-PAQ questionnaire (leisure-time, at work, and at home) | Trained personnel |
| EFRCV | Trained personnel. Weight: calibrated SECA electronic scale. Height: barefoot, Añó-Sayol S1B calibrated stadiometer. | Fasting state | Total cholesterol: enzymatic method (cholesterol esterase and cholesterol oxidase). HDL cholesterol: direct method after precipitation with phosphotungstic acid and Magnessium. Triglycerides: enzymatic method. | Average of 2 readings in the right arm, using a calibrated mercury sphygmomanometer (Diplomat, Riester 660/306). | Self-reported | Self-reported, validated ad-hoc questionnaire (compared against 7 daily logs of physical activity) | Self-reported |
| Control-MCC-SPAIN | Weight and height: self-reported. Waist circ.: trained personnel, 2-3 measurements, midpoint between the iliac crest and the lower ribs | Not available | Self-reported history of hypercholesterolemia. Participants from Madrid: primary care health records. Metabolomic analyses are currently being performed. | Self-reported history of hypertension. Participants from Madrid: primary care health records. | Trained personnel | Trained personnel. Baseline: ad-hoc questionnaire. 2020: IPAQ questionnaire | Self-reported, food frequency quest. |
| CORSAIB | Trained personnel. Weight: calibrated OMRON 711 electronic scale. Height: calibrated stadiometer. | Fasting state | Glucose: in DAX 72. Total cholesterol: enzymatic method. HDL cholesterol: direct method. Triglycerides: enzymatic method. | Average of 2 measurements in both arms, separated by 15 minutes, after several minutes in a seated position, with a calibrated OMRON tensimeter (Omron, Barcelona, Spain). | Trained personnel | Trained personnel, using the Spanish national health Survey questionnaire | Trained personnel |
| EMMA | Trained personnel, using calibrated scales and stadiometers in primary care centers. | Not available | Laboratory data: primary care clinical records (Local laboratory of Institut Català de la Salut) | Average of 2 measurements in both arms (to select the arm with the highest value), separated by 1 minute, after several minutes in a seated position, with a calibrated tensimeter. A highly discordant measurement (>5 mmHg) would imply a third measurement. | Trained personnel | Not available | Not available |
| ARTPER | Trained personnel, using calibrated scales. | Fasting state | Total, HDL, and LDL cholesterol; triglycerides, and glucose: from blood analysis (except when having a recent analysis within the last 12 months; in this case, information was extracted from medical records). | Average of 2 measurements (three measurements were taken and the highest was removed), in both arms (to select the arm with the highest value), after several minutes in a seated position, with a calibrated tensimeter. | Trained personnel | Trained personnel, using the short version of the Minnesota questionnaire | Not available |
| NEFRONA | Trained personnel, using calibrated scales | Fasting and non-fasting state | Laboratory data: hospital clinical records | Average of 3 measurements, left arm, after several minutes in a seated position, with a calibrated tensimeter. | Trained personnel | Not available | Not available |
| ILERVAS | Trained personnel, using calibrated scales, in light clothing. Height: in a calibrated stadiometer, barefoot. | Fasting state | Creatinine and cholesterol levels were assessed with the REFLOTRON Plus system (Roche Diagnostics, Germany) | Average of 3 measurements separated by 2 minutes, after 5 minutes in a seated position, with a calibrated Omron M6 Comfort tensimeter (Omron, Barcelona, Spain). | Trained personnel | Trained personnel, using the IPAQ questionnaire | Trained personnel |
| ASTURIAS | Trained personnel, using calibrated scales, in light clothing. Height: in a calibrated stadiometer, barefoot. | Fasting state | Glucose: hexokinase enzymatic method. Total cholesterol: cholesterol esterase and cholesterol oxidase enzymatic methods. HDL cholesterol: direct method using cholesterol esterase and oxidase enzymes modified with polyethylene glycol and α-cyclodextrin sulfatase. Triglycerides: enzymatic method using lipase glycerol kinase with colorimetric measurement. All assays were conducted using the Hitachi 747 system. | Average of 2 measurements separated by several minutes, after 30 minutes in a seated position, with a calibrated Omron Hem-703C tensimeter (Omron, Barcelona, Spain). | Trained personnel | Trained personnel | Not available |
| CDC de Canarias | Trained personnel. Waist circ.: midpoint between the iliac crest and the lower ribs | Fasting state | Glycemia and cholesterol in lipoproteins were measured with the Hitachi® 917 analyzer. | Average of 2 measurements separated by several minutes, after 5 minutes in a seated position. | Trained personnel | Trained personnel, using the Minnesota questionnaire | Trained personnel |
| HERMEX | Trained personnel. Weight: using calibrated SECA scales (ref.: 888 7021099), in light clothing. Height: in a calibrated SECA stadiometer (ref.: 222), barefoot. Waist circ.: midpoint between the iliac crest and the lower ribs | Fasting state | Glucose: hexokinase enzymatic method. Total cholesterol: cholesterol oxidase CHOD-PAP enzymatic method. HDL cholesterol: direct method. LDL cholesterol: direct, colorimetric-enzymatic method Triglycerides: GPO-PAP enzymatic method. | Average of 3 measurements separated by 2 minutes, after 5 minutes in a seated position, using a calibrated OMRON HEM 907 automatic tensimeter (Omron, Barcelona, Spain). | Trained personnel | Trained personnel, using the Minnesota questionnaire | Trained personnel |
| HORTEGA | Trained personnel | Non-fasting state (~3h) | Lipid profile: lipid particle profiles were quantified using the LIPOSCALE NMR methods (Biosfer Teslab, Reus, Spain). | Average of 3 measurements, after several minutes in a seated position, using a calibrated OMRON Hem-711C automatic tensimeter (Omron, Barcelona, Spain). | Trained personnel | Trained personnel, using their own validated questionnaire | Trained personnel |
| NAVARRA 93 | Trained personnel. Weight: calibrated SECA scales (ref.: 760), in light clothing. Height: in a calibrated KAWE stadiometer (ref.: 222), barefoot. | Fasting state | Glucose: hexokinase enzymatic method. Total cholesterol: cholesterol oxidase CHOD-PAP enzymatic method. HDL cholesterol: oxidase CHOD-PAP, direct method. Triglycerides: GPO-PAP enzymatic method. | Average of 2 measurements, right arm, after several minutes in a seated position, using a calibrated Hawksley tensimeter | Trained personnel | Not available | Not available |
| EPIC-Navarra | Trained personnel. Weight: calibrated scale. Height: barefoot, calibrated stadiometer. Waist circumference: as the narrowest circumference of the torso (natural waist) and at the midpoint (halfway between the iliac crest and the lower ribs) | Fasting and non-fasting | Glucose, creatinine, triglyceride, total and HDL cholesterol were measured by enzymatic colorimetric tests. | Not available | Self-reported | Self-reported, using the validated EPIC-PAQ questionnaire (leisure-time, at work, and at home) | Trained personnel |
| RIVANA | Trained personnel. Weight: calibrated scale. Height: barefoot, calibrated stadiometer. Waist circumference: as the narrowest circumference of the torso (natural waist) and at the midpoint (halfway between the iliac crest and the lower ribs) | Fasting state | Glucose: hexokinase enzymatic method. Total cholesterol: cholesterol oxidase CHOD-PAP enzymatic method. HDL cholesterol: direct method. Triglycerides: GPO-PAP enzymatic method. | Average of 3 measurements separated by 5 minutes, in both arms (the one with a highest value was selected), after several minutes in a seated position, using a calibrated OMRON M4-1 automatic tensimeter (Omron, Barcelona, Spain). | Trained personnel | Trained personnel, using the Minnesota questionnaire | Trained personnel (MEDAS score) |
| RECCyL | Trained personnel | Fasting state | Glucose and lipid profile: enzymatic colorimetric methods. | Average of 2 measurements separated by 2 minutes, in both arms, after 5 minutes in a seated position, using a calibrated tensimeter. | Self-reported | Self-reported (number of hours of physical activity per week) | Not available |
| PREDIMERC | Trained personnel. Weight: Tefal Lotus Sensitive calibrated scales. Height: SOEHNLE calibrated stadiometer. | Fasting state | Glucose: glucose oxidase method. Total cholesterol: cholesterol esterase and cholesterol oxidase. HDL cholesterol: direct method. Triglycerides: lipase/glycerol kinase method. All laboratory tests were conducted using the Modular P800 Roche Diagnostics | Average of 3 measurements separated by 1 minute, after 5 minutes in a seated position, using a calibrated OMRON HEM-705-CP_II automatic tensimeter (Omron, Barcelona, Spain) | Self-reported | Self-reported (intensity and frequency of several activities) | Self-reported |
| IBERICAN | Trained personnel, using calibrated scales and stadiometers available in primary care | Fasting state | Measurements were performed in the Reference Laboratory of each Health Center. | Average of 2 measurements after 5 minutes in a seated position | Self-reported | Self-reported (categories: sedentary lifestyle, low moderate exercise, moderate exercise (> 60 min/day), play sport regularly) | Trained personnel |
| AEGIS | Trained personnel. Weight: calibrated Roman scale. Height: barefoot, calibrated stadiometer. Waist circumference: as the narrowest circumference of the torso (natural waist) and at the midpoint (halfway between the iliac crest and the lower ribs) | Fasting state | Glucose: hexokinase enzymatic method. HDL cholesterol: direct method. Triglycerides: enzymatic method. Determinations were performed in an ADVIA 2400 autoanalyzer (Siemens). | Average of 2 measurements separated by 5 minutes, left arm, after 30 minutes in a seated position, using a calibrated OMRON automatic tensimeter (Omron, Barcelona, Spain). A highly discordant measurement (>5 mmHg) would imply a third measurement. | Self-reported | Self-reported, using the IPAQ questionnaire | Self-reported |
| DI@BET.ES-EUSKADI | Trained personnel, using SOEHNLE calibrated scales. Waist circ. was measured at the level of the navel. | Fasting state | Glucose: hexokinase enzymatic method. Total cholesterol: cholesterol oxidase enzymatic method. HDL cholesterol: direct method. Triglycerides: glycerol phosphate oxidase enzymatic method. Analyses performed in an Architect C8000 Analyzer (Abbott Laboratories SA, Madrid, Spain). | Average of 2 measurements separated by 2 minutes, after several minutes in a seated position, with a calibrated tensimeter Hem-703 C (Omron, Barcelona, Spain).  . | Trained personnel | Trained personnel, using the IPAQ questionnaire | Trained personnel |
| CARGENCORS | Trained personnel, using calibrated scales and stadiometers. | Fasting and non-fasting | Laboratory data: hospital and primary care clinical records | Average of 2 measurements  using an automatic sphygmomanometer after 5 minutes of rest. | Self-reported | Not available | Not available |
| GCAT | Trained personnel, using calibrated scales. | Fasting and non-fasting | Laboratory data: hospital and primary care clinical records  Metabolome analyses are underway. | Average of 2 measurements separated by 2 minutes, in a seated position, using a calibrated tensimeter. | Self-reported | Self-reported, based on using the IPAQ questionnaire | Self-reported |
| RICARTO | Trained personnel, using calibrated scales and stadiometers. Waist circ. was measured at the level of the navel. | Fasting state | No details available | Average of 3 measurements separated by 1 minute, after 5 minutes in a seated position, with a calibrated tensimeter OMRONHEM-907 (Omron, Barcelona, Spain). | Trained personnel | Trained personnel (one simple question) | Trained personnel |

**Supplemental Table 4.** Percentage of valid values per cohort and variable.

|  | Sample  size | Age  (%) | Sex  (%) | Origin  (%) | Education  (%) | Tobacco  (%) | HT  (%) | SBP  (%) | DBP  (%) | HR  (%) | Diabetes  (%) | Glucose  (%) | TC  (%) | HDL-C  (%) | LDL-C  (%) | Trig.  (%) | Chol.tt (%) | BMI  (%) | Creat.  (%) |
| --- | --- | --- | --- | --- | --- | --- | --- | --- | --- | --- | --- | --- | --- | --- | --- | --- | --- | --- | --- |
| REGICOR | 11,632 | 100 | 100 | 65 | 99 | 99 | 100 | 100 | 100 | 99 | 100 | 97 | 97 | 97 | 95 | 97 | 98 | 99 | 29 |
| ACRISC | 743 | 100 | 100 | 0 | 99 | 100 | 100 | 100 | 100 | 98 | 100 | 99 | 99 | 99 | 97 | 100 | 100 | 100 | 0 |
| BARCOS | 473 | 100 | 100 | 98 | 0 | 98 | 100 | 31 | 31 | 1 | 100 | 81 | 78 | 56 | 56 | 78 | 91 | 77 | 87 |
| AWHS | 5,670 | 100 | 100 | 0 | 0 | 99 | 100 | 100 | 100 | 100 | 100 | 100 | 100 | 100 | 97 | 100 | 99 | 100 | 100 |
| SALMANTICOR | 1,993 | 100 | 100 | 100 | 100 | 100 | 100 | 99 | 99 | 98 | 99 | 55 | 54 | 55 | 50 | 55 | 90 | 100 | 55 |
| DI@BET.ES | 5,419 | 100 | 100 | 100 | 93 | 93 | 100 | 92 | 92 | 0 | 100 | 92 | 87 | 87 | 87 | 87 | 100 | 92 | 87 |
| PIZARRA | 2,089 | 100 | 100 | 32 | 31 | 100 | 100 | 100 | 100 | 100 | 100 | 99 | 90 | 87 | 57 | 90 | 100 | 100 | 90 |
| PREDAPS | 2,022 | 100 | 100 | 100 | 99 | 100 | 100 | 100 | 100 | 100 | 100 | 100 | 100 | 99 | 98 | 100 | 31 | 100 | 100 |
| DRECE | 3,465 | 100 | 100 | 0 | 40 | 98 | 100 | 99 | 99 | 0 | 100 | 100 | 100 | 100 | 100 | 98 | 92 | 99 | 100 |
| ENRICA | 13,018 | 100 | 100 | 98 | 100 | 100 | 100 | 94 | 94 | 94 | 100 | 99 | 99 | 100 | 98 | 100 | 29 | 93 | 99 |
| EPIC-Granada | 7,864 | 100 | 100 | 0 | 99 | 100 | 100 | 28 | 28 | 27 | 100 | 0 | 11 | 11 | 10 | 11 | 0 | 100 | 10 |
| EPIC-Gipuzkoa | 8,417 | 100 | 100 | 0 | 100 | 100 | 100 | 18 | 18 | 19 | 100 | 0 | 16 | 16 | 16 | 16 | 0 | 100 | 15 |
| EPIC-Murcia | 8,515 | 100 | 100 | 0 | 100 | 100 | 100 | 32 | 32 | 32 | 100 | 0 | 16 | 16 | 16 | 16 | 0 | 100 | 16 |
| EFRCV | 3,089 | 100 | 100 | 0 | 100 | 100 | 100 | 100 | 100 | 0 | 98 | 0 | 78 | 74 | 0 | 78 | 100 | 100 | 0 |
| Control-MCC-SPAIN | 4,077 | 100 | 100 | 100 | 99 | 100 | 100 | 5 | 5 | 0 | 100 | 2 | 5 | 4 | 4 | 5 | 96 | 94 | 5 |
| CORSAIB | 1,685 | 100 | 100 | 0 | 100 | 100 | 100 | 100 | 100 | 100 | 100 | 100 | 100 | 99 | 93 | 100 | 100 | 99 | 0 |
| EMMA | 35,175 | 100 | 100 | 0 | 0 | 100 | 100 | 100 | 100 | 0 | 100 | 100 | 100 | 100 | 100 | 100 | 100 | 100 | 0 |
| ARTPER | 3,736 | 100 | 100 | 100 | 95 | 100 | 100 | 100 | 100 | 98 | 100 | 99 | 99 | 100 | 99 | 99 | 100 | 100 | 0 |
| NEFRONA | 3,004 | 100 | 100 | 0 | 0 | 100 | 100 | 100 | 100 | 0 | 100 | 99 | 97 | 88 | 87 | 87 | 100 | 100 | 74 |
| ILERVAS | 8,330 | 100 | 100 | 0 | 0 | 100 | 100 | 100 | 100 | 0 | 100 | 0 | 100 | 32 | 31 | 32 | 100 | 100 | 100 |
| ASTURIAS | 1,034 | 100 | 100 | 0 | 0 | 0 | 100 | 100 | 100 | 0 | 100 | 100 | 100 | 99 | 98 | 100 | 0 | 100 | 100 |
| CDC Canarias | 7,160 | 100 | 100 | 92 | 94 | 94 | 100 | 99 | 99 | 98 | 94 | 93 | 93 | 93 | 93 | 93 | 28 | 99 | 0 |
| HERMEX | 2,833 | 100 | 100 | 100 | 99 | 100 | 100 | 100 | 100 | 100 | 100 | 100 | 100 | 100 | 97 | 100 | 100 | 100 | 100 |
| HORTEGA | 1,475 | 100 | 100 | 0 | 99 | 100 | 100 | 99 | 99 | 0 | 100 | 0 | 100 | 99 | 100 | 100 | 100 | 96 | 100 |
| NAVARRA 93 | 1,480 | 100 | 100 | 99 | 100 | 100 | 100 | 100 | 100 | 100 | 100 | 99 | 99 | 99 | 100 | 100 | 12 | 100 | 0 |
| EPIC-Navarra | 7,953 | 100 | 100 | 0 | 99 | 100 | 100 | 0 | 0 | 0 | 100 | 0 | 17 | 17 | 16 | 17 | 0 | 99 | 16 |
| RIVANA | 4,164 | 100 | 100 | 100 | 100 | 100 | 100 | 100 | 100 | 98 | 100 | 100 | 100 | 100 | 99 | 100 | 100 | 100 | 100 |
| RECCyL | 3,761 | 100 | 100 | 99 | 0 | 99 | 100 | 99 | 99 | 0 | 100 | 98 | 99 | 97 | 93 | 99 | 98 | 89 | 9 |
| PREDIMERC | 2,268 | 100 | 100 | 99 | 100 | 100 | 100 | 100 | 100 | 100 | 100 | 100 | 100 | 100 | 100 | 100 | 100 | 100 | 100 |
| IBERICAN | 8,035 | 100 | 100 | 0 | 100 | 99 | 100 | 100 | 100 | 100 | 100 | 100 | 100 | 93 | 93 | 100 | 100 | 100 | 98 |
| AEGIS | 1,486 | 100 | 100 | 100 | 100 | 100 | 100 | 100 | 100 | 100 | 100 | 100 | 100 | 100 | 99 | 100 | 100 | 100 | 100 |
| DI@BET.ES-EUSKADI | 567 | 100 | 100 | 100 | 100 | 100 | 100 | 100 | 100 | 0 | 100 | 100 | 99 | 99 | 97 | 100 | 100 | 100 | 100 |
| CARGENCORS | 2,838 | 100 | 100 | 0 | 0 | 100 | 100 | 99 | 99 | 0 | 100 | 98 | 93 | 87 | 84 | 91 | 0 | 97 | 97 |
| GCAT | 19,187 | 100 | 100 | 100 | 99 | 95 | 100 | 100 | 100 | 100 | 100 | 66 | 66 | 59 | 54 | 60 | 100 | 100 | 65 |
| RICARTO | 1,975 | 100 | 100 | 0 | 100 | 100 | 100 | 100 | 100 | 100 | 100 | 100 | 99 | 99 | 99 | 100 | 100 | 100 | 100 |
| All | 196,632 | 100 | 100 | 41 | 67 | 98 | 100 | 84 | 84 | 49 | 100 | 70 | 79 | 74 | 72 | 75 | 72 | 99 | 46 |

*BMI*: body mass index; *Chol.tt.*: treated with cholesterol-lowering drugs; *Creat*.: creatinine; *DBP*: diastolic blood pressure; *HDL-C*: HDL cholesterol; *HR*: heart rate; *HT*: hypertension; *LDL-C*: LDL cholesterol; *SBP*: systolic blood pressure; *TC*: total cholesterol; *Trig*.: triglycerides; *Univ*.: university.

**APPENDIX**

**Appendix I.** List of investigators and scientific collaborators of the CORDELIA cohorts.

**REGICOR.** J. Marrugat, R. Elosua, I.R. Dégano, I. Subirana, Á. Hernáez, A. Camps-Vilaró, S. Polo-Alonso, H. Tizón, M. Cainzos-Achirica.

**ACRISC**. M. Grau, D. Zomeño.

**BARCOS**. D. Ovejero, X. Nogués, M. Pineda, N. Garcia-Giralt.

**AWHS**. J.A. Casasnovas, V. Alcaide, F. Civeira, E. Guallar, B. Ibañez, J. Jiménez Borreguero, M. Laclaustra, M. León, J.L. Peñalvo, J.M. Ordovás, M. Pocovi, G. Sanz, V. Fuster.

**SALMANTICOR**. P.L. Sánchez, P.I. Dorado-Díaz, C. Sánchez, A. Romero-Furones, J.I. Melero-Alegría, M. Cascón, P.P. Vara, S. Cascón, F. Pérez-Escanilla, J. Hernández-Hernández, B. Blazquez, J.M. de Dios, J.M. Hernández, C. Sánchez-Pablos, I. Santolino, M.C. Ledesma, P. Muriel.

**DI@BET.ES.** G. Rojo-Martínez, S. Valdés, N. Colomo, C. Maldonado W Oualla-Bachiri, S García-Serrano, E. García Escobar, A. Lago-Sampedro, A. Calle, L. Castaño, S. Gaztambide, I. Urrutia, J.F. Chaves, J. Girbés, E. Delgado, J. Franch-Nadal, A. Goday, E. Ortega, F. Soriguer. Di@bet.es group full list: <https://www.sediabetes.org/cientifico-y-asistencial/investigacion/proyectos-de-investigacion/estudio-dibet-es/>.

**PIZARRA.** G. Rojo-Martínez, S. Valdés, N. Colomo, C. Maldonado, W. Oualla-Bachiri, S. García-Serrano, E. García Escobar, A. Lago-Sampedro, M.S. Ruiz de Adana, I. Esteva, M.C. Almaraz, F. Linares, F. Soriguer.

**PREDAPS**. B. Benito-Badorrey, M. Birules-Pons, M.I. Bobé-Molina, J.J. Cabré-Vila, G. Cuatrecasas-Cambra, J. Franch-Nadal, J.C. González-Pastor, F. López-Simarro, R. López- López, M. Mata-Cases, X. Mundet-Tudurí, T. Mur-Marti, R. Pujol-Martínez, A. Rodríguez- Poncelas, L. Romera-Liébana, P. Roura-Olmeda, I. Ruiz-Tamayo, M. Villaro-Gabarros, R.M. de Miguel-Pérez.

**DRECE**: J.A. Gutiérrez Fuentes, J.A. Gómez Gerique, A. Gómez de la Cámara, M. A. Rubio Herrera, P. Cancelas, C. Jurado, A. García, J. del Campo, S. Campiña, A. Rueda, A. Avellaneda, R. Montero, C. Moreno, M.D. Ballesteros, B. Martín Ballesteros, R. García Sardina, C. Lasa Unzúe, J.D. García Díaz, T. Montoya, A. Porres, E. Juncadella, I. García, E. Melús Palazón, M.J. Morales Gregorio, P. Pitarque Cargallo, E. Mayayo Castillejo, I. González Gómez de Segura, M.I. Sancho Giner, A.M. Aznárez García, F. Ibáñez García, M.E. Marco Gayarre, P. Sebastián Villán, E. Muñoz Novella, M.A. Montañés Gracia, S. Murciano González, V. Peg Rodríguez, M. Martell, A. Aguiar Bautista, V. del Rosario Sánchez, M.C. Gómez Medina, M. Martel López, D. Ruano López, R. Sáenz Guallar, A. Abos Zueco, J. Pastor Espinosa, J.J. Berlanga Rubio, A. García García, M.E. Estopiñán, B. Altaba Sanz, I. Castellano Juste, C. Burgues Valero, C. Gil Muñoz, J.A. Malagón Chaves, A. Mora Belda, M. Badía Savidó, S. Puerto Bacete, M.D. Luna Albero, A. García Barrientos, A. Sarrió Sanchos, E. Calatayud Climent, E. Hernández Hernández, C. Corpas, E. Rodríguez Extremera, A. Cabero Alemán, M.A. López de la Llama, O. Franco Pizarro, I. Pérez Suárez, R. Provencio Hernando, F.J. Peiró Cifuentes, M. Cruz, A. Gómez, C. Cabezas, J.A. Esparza Salcedo, G. Martín Gracia, F. Sancho Durán, J. Boned Izued, J. Codes Gómez, M.C. Ortega Calleja, R. Alonso, R. Martínez Subiela, J.R. Lorca Serralta, A. Sancho, M.A. Díez García, C. Hernández Sanjosé, M.A. López Dolado, I.M. Socias Buades, M. Campo Vázquez, M. Garáu Miquel, F. Ramón Roselló, M.J. Barea Mestre, M. Barceló Morey, S. Farrouf, A. Peña, A. Morales, M.J. Avellana, J.B. Gómez Castaño, J.L. Molina Molina, A. Villa Salmerón, F.J. Ortiz Martínez, M. Semipiel Espín, M.L. García Navarro, F. Vaquero Garcés, T. Seco Pérez, M.J. Pastor Sanbruno, C. Escolar Carrión, S. Pelayo Bando, R. de Silva Rodríguez, C. Palacios Villanueva, A. Ramos Corpas, C. Muñoz Lanzas, P. Carrero Fernández, E. Pérez Calzada, M.V. Alonso Pérez de Ágreda, M.Y. del Campo Ciruelos, M.T. Díaz Benito, M.A. González Ramos, C. González Ramos, C. Sánchez Arce, T. Casaseca Calvo, A. Sanz Montenegro, S. Cantalapiedra Ortega, M.J. Nadal Blanco, O. Pascual Gil, M. Sáez Pomares, J.M. González Vaquero, M. García Sala, J. Fluixá Sendra, J.A. Azorín Puche, M.J. Muñoz Reig, N. Domenech Climent, C. Amezqueta Goñi, A. Eraso Zabalza, M.C. Gutiérrez Pardo, T. Albert Amorós, Campello Pérez, V.M. Conca Pérez, M.L. Álvarez Terres, C. Pérez Valero, M. Bri Grau, F. Rodríguez Ruiz, A. García García, A. Carreras López, M. Marín Collado, C. de Gregorio y Bernardo, J.F. Ruiz Sánchez, J.A. López Grau, J. Quinto Juan, S. Damián Sanmartín, A. Sánchez Hernández, J.M. Amorós Toral, F. Tari Macía, R. Antón Tortosa, M. Fernández Carreira, J. Cachón, F. Almagro, E. Yetano Larrazábal, E. Intxaurza Hernández, J. Caballero, C. Rodríguez Muñoz, E.P. Rodríguez Naranjo, J.M. Fernández Carreira, L. Gómez Esmóris, J.M. García, S. Aranda Sánchez, M.L. Sánchez de Andrés, M. Borrego, R. Corona Muñoz, M.C. Bárcena, F. Puntes, T. Alonso, M. Fuentes, J. Fortea, F. López Simarro, S. Miravet Jiménez, J. Fortea López, I. Verges Macario, D. Voces García, C. Morales Martínez, M. I. del Cura González, C. Reverte Asuero, J.I. Sedano García, E. Angulo Vallejo, M. A. Martínez Solórzano, M. Santos Lago, R. Pérez Madrazo, J.L. Ortiz de Pinedo García, E. Picasso Gallego, J. Velasco Villaba, J. Franco Girado, M. Serrano Aranda, J.M. Díaz Blanco, R. Alfaro Gómez, M. Gómez Ríos, L.L. Rodón Palomino, F. Moreno Castro, A. Naharro Hernández, M. Ortega Calvo, M. Ochoa Casteleiro, I. Hermoso Cano, C. Gordillo, G. Alfaro, P. Rey, T. Lerma, F. San Juan García, A. Cruz Macías, S. Vilariño Román, J. Isasia Ballestero, M.J. Martín Martín, M. Monasterio Bazán, C. Alonso Canosa, Y. Zorraquino Muñoz, L.E. Gómez Rodríguez, L.A. Serrano Cumplido, J. Antón Ortega, J. del Río Fernández, L. Uribe-Etxebarría García, N. López Miguel, E. Borobio del Campo, J. Marín Vieites, A. García García, D.I. García de las Heras, M. Aragón Fierro, M.J. Castellanos Alonso, M. Pellitero Espina, C. Jurado, M.A. Ortega Gómez, A. Bárcena Marugán, J. Villamayor Suárez, M. Poza Bravo, G. Arroyo Pérez, A.L. Chamorro, P. Muñoz Mateos, I. Redondo Cuesta, J.J. Peris Ricart, C. Monteagudo Suárez, M. Pérez Egido, L.M. Fontenla Devesa, M.C. Luna Barrós, M. C. Paz Silva, R. Rubianes Soto, M.D. Durán Pereira, R.D. Martínez Meijide, N. Silva García, M.A. Abeti Sarasa, M. Buttini, E. Llort Blasco, I. Forcada Higuet, C. Jurado Nieto, M. Rodríguez Bobillo, D. Gómez Gallardo, A. Ponce Feria, M.C. Velicia Peña, M. Domínguez Sardiña, J. Mosquera Nogueira, M. Rodríguez Ríos, V.J. Diéguez Pereira, C. Gabián Pereira, M. Velhas Pereira, X.M. Parente Mojón, J.A. Río Orgueira, C. Cruces Artero, M.A. Rionegro López, F. Bestar, J. Tera Donoso, C. Lamas, H. Cardona Castellano, M.D. Carrascosa Ferrera, J. Marrero Brito, J. Horno Delgado, L. Alquezar Labad, B. Gargallo, J. Castiella Herrero, M.A. de Mingo, S. Hernández, P. Ferrando, D. Lora, J. de la Cruz, P. Magán, M. Menénedez Orenga, J.C. Calvo Fernández, F. Rivas Botana, M. Fernández del Río, A. Reguera Barba, M.T. Díez, S. Aparicio García, D.V. Palomo Gómez, M. Bacariza Cortiñas, P. González Aido, A. Álvarez Caride.

**ENRICA**. F. Rodríguez-Artalejo, A. Graciani, P. Guallar-Castillón, L.M. León-Muñoz, C. Zuluaga, E. López-García, J.L. Gutiérrez-Fisac, J.M. Taboada, T. Aguilera, E. Regidor, F. Villar-Álvarez, J.R. Banegas.

**EPIC-Granada.** M.J. Sánchez Pérez, M. Rodríguez Barranco, E. Molina Montes, D. Petrova, N.F. Fernández Martínez, J.M. Gálvez Navas.

**EPIC-Gipuzkoa**. P. Amiano, A. Jimenez-Zabala, O. Mokoroa-Carollo, L. Alvarez-Gerriko, E. Abilleira-Cillera, A. Aizpurua, A. Llorente-Aginagalde

**EPIC-Murcia**. M.D. Chirlaque López, J.M. Huerta Castaño, J.H. Gómez Gómez, C. Santiuste de Pablos, S.M. Colorado Yohar, N. Cabrera Castro.

**EFRCV**. J.M. Huerta Castaño, M.D. Chirlaque López, D. Pérez Flores, D. Salmerón Martínez.

**Control-MCC-Spain**. M. Pollán, N. Aragonés, A. Sierra, M. Kogevinas, G. Castaño-Vinyals, V. Moreno, D. Casabonne, E. Ardanaz, M. Guevara, P. Amiano, V. Martín, A. Tardón, M.D. Chirlaque, J. Alguacil, T. Dierssen, I. Gómez Acebo, A. Molina, J.J. Jiménez Moleón, R. Marcos-Grajera, P. Fernandez-Navarro.

**CORSAIB**. F. Rigo, G. Frontera, F. Unda-Villafuerte, M. Truyol-Mas, G. Galve-Dolz, and 50 Medical Doctors of the Mallorca region (fieldwork).

**EMMA**. R. Martí Lluch, M. Quesada Sabaté, E. Balló Peña, R. Ramos Blanes.

**ARTPER**. M.T. Alzamora, R. Forés, J.M. Baena-Díez, G. Pera, A. Heras, M Valverde M. Sorribes M. Vicheto, M.D Reina, A. Sancho, C. Albaladejo, J Llussà,C. Vela, A. Heras,J Ingla, J. Alegre, B. López, M Corroto,J Milozzi, M Isnard, M. To the members who have been part of the ARTPER study group and to all members of the Primary Care Teams of Barcelonès Nord i Maresme area and Barcelona city who participated in the inclusion of participants in the cohort.

**NEFRONA**. J.M. Valdivielso, M. Bermúdez, M. Bozic.

**ILERVAS**. J.M. Valdivielso, M. Bermúdez, M. Bozic, E. Castro, C. Farràs, V. María, M. Elias, T. Molí, C. Domínguez, N. Nova, A. Prunera, N. Sans, M. Soria, F. Pons, R. Senar, M. Agustí, C. García, M. Guerrero. The team would also like to thank Fundació Renal Jaume Arnó, and the Primary Care teams of the province of Lleida.

**ASTURIAS**. P. Botas Cervero, E. Delgado Álvarez, E. Menéndez Torre, S. Valdés Hernández, J. Ares Blanco. The team would also like to thank the Primary Care teams of the selected basic health zones, the INSALUD Studies Cabinet, S. Rozada, I. Huerta, J.R. Quirós, the Endocrinology group of Asturias, and A. Cobo Irusta.

**CDC de Canarias.** A. Cabrera de León, D. Almeida González, M. del Cristo Rodríguez Pérez, I. Marcelino Rodríguez, F.J. Cuevas Fernández, A. León-Hernández.

**HERMEX**. F.J. Félix-Redondo, D. Fernández-Bergés, P. Álvarez-Palacios, V. Tejero, A.B. Hidalgo, Y. Morcillo, M.J. Zaro, L. Lozano, J.F. Pérez-Castán, N.R. Robles, J.F. Macias, B. Cancho, J. Villa, F. Buitrago, J.M. Ramírez, L. Palomo, H. Sanz, M. Grau, J. Vila, J.M. Baena-Diez, I. Subirana, V.A. Aparicio, A. Soriano-Maldonado, M. Flor-Alemany, J.J. Garrido. The team would also like to thank: G. Cebrián, V. Gallego, A. Gómez, E. Villanueva, F.J. Valadés.

**HORTEGA.** J.C. Martín Escudero, F. Simal Blanco, F.J. Mena Martín, J. Bellido Casado, D. Arzua Mouronte, F. Pinacho, I. González Melgosa, M. Tabuyo Pizarro, J.L. Carretero Ares, A.A. Álvarez Hurtado, B. Alonso Gallego, A. Noriega Migues, J. Tasende Mata, M. Herrero Baladrón, R. Frutos Llánes, I. Sánchez Lite, M.G. Pérez Paredes, F. Pérez Fernández, M. Alegre Climent, R.N. Pérez Martín, J. Rodríguez Núñez, R. López Izquierdo, C.A. Burgos Díez, R. de Prado Berrocal, I. González Manzano, T. Alonso Ares, S. Otero de la Torre, E.L. de Sande Nacarino, M. Pineda Alonso, L.S. Briongos Figuero, A. Nieto de Pablos, P. Celis Sanchez, A. Dueñas Laita, A. Mayo Iscar, F.J. Castrodeza Sanz, V. Herreros Fernández, F.J. Chaves, M. Tellez-Plaza, J. Redón Mas, A.B. García García.

**NAVARRA 93.** M.J. Guembe Suescun, E. Ardanaz, C. Moreno Iribas, A. Barricarte, M.J. Urra, C. Sayón-Orea. The team would also like to thank the cohort participants, Navarre Health Service/Osasunbidea, the University Hospital of Navarra, the Department of Health of the Government of Navarra, the Instituto de Salud Pública y Laboral de Navarra, and the Instituto de Investigación Sanitaria de Navarra (IdisNa).

**EPIC-Navarra**. M. Guevara, E. Ardanaz, J. Delfrade, A. Barricarte, C. Moreno-Iribas.

**RIVANA**. M.J. Guembe Suescun, J.J. Viñes Rueda, P. González Diego, C. Moreno Iribas, I. Sobejano Tornos, C. Amézqueta Goñi, A. Grijalba Uche, M. Artazcoz, J. Barba Cosials, J. Díez Martínez, E. Martínez Vila, P. Irimia, D. Guerrero Setas, E. Los Arcos Lage, J. Berjón Reyero, E. Toledo Atucha, C. Sayón Orea, M.J. Urra, A. Martínez Hernandez, C.I. Fernández Lázaro, and the researchers of the RIVANA group. The team would also like to thank the cohort participants, Navarre Health Service/Osasunbidea, the University Hospital of Navarra, the Department of Health of the Government of Navarra, the Foundation Miguel Servet, the Navarrabiomed Biobank, and the fieldwork professionals of the study.

**RECCYL**. T. Vega Alonso, J.E. Lozano Alonso, R. Álamo Sanz, S. Lleras Muñoz, M.P. Rodríguez Martín, J. Cordero Guevara, J. Naveiro Rilo, M.J. Barrenechea Fernández, M. González García, A. Escribano, B. de la Hoz García, C. Andrés Gonzalo, R. Arquiaga Thireau, T. Martínez Campos, L. Ledesma Santiago. A. Ordax Díez, I. Martínez Pino.

**PREDIMERC**. E. Gil Montalbán, E. Donoso Navarro, B. Zorrilla Torras, H. Ortiz Marrón, M. Martínez Cortés, M.D. Esteban Vasallo, L.M. Blanco, F. Domínguez-Berjón. A. M Gandarillas Grande. The team would also like to thank the participants and DEMOMÉTRICA.

**IBERICAN**. Scientific Committee: A. Barquilla García, Á. Díaz Rodríguez, C. Escobar Cervantes, F. J. Alonso Moreno, J. Vergara Martín, J. J. Badimón, J. Polo García, L. Rodríguez Padial, M. Á. Prieto Díaz, R. Vidal Pérez, S. Cinza Sanjurjo, S. Miravet Jiménez, S. Velilla Zancada, J. R. Banegas, V. Martín Sánchez, V. Pallares Carratalá, A. Segura Fragoso, R. M. Micó Pérez. Collaborators: A. López Téllez, J. Vergara Martin, M. D. L. Á. Ortega Osuna, J. Lorente Serna, Á. Domínguez Requena, F. Herrero Collado, R. Á. Carrascal Garrido, M. R. Herrera Lozano, B. Ortiz Oliva, C. Pérez Ibáñez, M. J. Cruz Rodríguez, S. Abad Sánchez, I. Santana Martínez, R. Sánchez Jordán, J. M. Ramos Navas-Parejo, J. M. Ramírez Torres, J. M. Beltrán Poveda, M. A. De Cruz Benayas, J. I. Esturo Alcaine, F. Leiva Cepas, J. L. Carrasco Martín, E. García Criado, L. Ginel Mendoza, J. C. Aguirre Rodríguez, J. Acevedo Vázquez, J. G. García Ballesteros, P. Agüera Moreno, J. M. Ignacio Expósito, N. Carrillo Peñas, C. M. Abad Faya, A. M. Almagro Duque, R. Torrescusa Camisón, P. Menéndez Polo, M. Peña García, C. López Fernández, A. Estepa Torres, E. Loizaga González, L. García Matarín, E. J. Gamero De Luna, J. Benítez Rivero, M. J. Gómez González, C. Gómez Montes, J. M. González Barranco, J. Ramírez Vizcaíno, M. Á. Miranda Sánchez, E. Trillo Calvo, C. Bayod Calvo, S. Larripa De La Natividad, E. Jiménez Marín, A. C. Navarro Gonzalvo, A. P. Martínez Barseló, I. Peña León, Á. González Pérez, L. Mahulea, M. J. Pérez Martínez, A. Piera Carbonell, M. Alonso Fernández, M. M. Rueda Cuadrado, R. Abad Rodríguez, J. M. Álvarez Cabo, R. Sánchez Rodríguez, E. M. Cano Cabo, A. Romero Secin, N. Dopico Sanesteban, M. Á. Prieto Díaz, J. J. García Fernández, F. García Romanos, A. Moreno González, M. L. Amengual Sastre, S. Martínez Palli, J. A. Ramón Bauza, J. Ortiz Bolinches, C. Fernández Fernández, M. I. Orlandis Vázquez, A. Sanchis Mezquita, F. Unceta Aramburu, J. F. Peiró Morant, A. Moyá Amengual, M. Seguí-Díaz, J. F. Zuazagoitia Nubla, A. Echevarría Ituiño, G. Mediavilla Tris, M. C. Noriega Bosch, E. González, M. L. Ruiz Macho, R. Sendino Del Olmo, A. Olagorta De Prado, A. López De Viñaspre Muguerza, J. Iturralde Iriso, M. R. Virtus Iñurrieta, I. Godoy García, F. Rubio Sevillano, M. I. González González, M. Pérez Souto, R. De León Contreras, I. Almería Diez, V. M. Mirabal Sánchez, F. J. Escobar Lavado, N. Sánchez Hernández, J. L. Alonso Jerez, R. Koch, N. Ramírez Mendoza, H. Suárez Hernández, F. J. Morales Escobar, E. L. Gutiérrez Fernández, F. A. Mantecón, A. B. García Garrido, A. Vélez Escalante, L. Alonso Rentería, J. Sainz Jiménez, G. Pombo Alles, J. A. Divisón Garrote, P. Martínez Sotodosos, J. A. Vivancos Fuster, M. García Palencia, S. González Ballesteros, A. C. Gil Adrados, A. González Cabrera, M. Á. Babiano Fernández, J. J. Criado Álvarez, P. Torres Moreno, F. J. Arribas Aguirregaviria, A. Sahuquillo Martínez, R. Piedra Castro, C. Santos Altozano, L. González Tarrio Polo, P. Valiente Maresca, R. Mota Santana, N. E. Terrero Ledesma, N. Garrido Espada, F. J. Alonso Moreno, G. D. Rosa Zambrana Calvi, C. De Castro Mesa, A. M. De Santiago Nocito, C. Lozano Suárez, J. L. Gutiérrez Montero, J. I. López Gil, M. D. Fernández Ortega, M. E. Roncal, M. A. López Serrano, N. E. Adrián De La Fuente, B. Angulo Fdez. De Larrea, N. Cubelos Fernández, G. L. Ferreiro Gómez, D. Gómez Rodríguez, S. De Abajo Olea, J. J. León Regueras, C. M. Gallego Nieto, D. Vázquez Mallada, M. D. L. O. Gutiérrez García, P. Baz Rodríguez, J. I. Ferradal García, B. D. De Román Martínez, A. Arconada Pérez, O. M. Atoui, J. L. Gutiérrez Montero, J. I. López Gil, M. D. Fernández Ortega, M. E. Roncal, M. A. López Serrano, N. E. Adrián De La Fuente, B. Angulo Fdez. De Larrea, N. Cubelos Fernández, G. L. Ferreiro Gómez, D. Gómez Rodríguez, S. De Abajo Olea, J. J. León Regueras, C. M. Gallego Nieto, D. Vázquez Mallada, M. D. L. O. Gutiérrez García, P. Baz Rodríguez, J. I. Ferradal García, B. D. De Román Martínez, A. Arconada Pérez, O. M. Atoui, G. Rovira Marcelino, D. E. Fernández Valverde, R. Rodó Bernadó, N. Najih, J. M. Diéguez Parra, M. R. Benedicto Acebo, M. L. Bravo Vicien, A. Mostazo Muntané, B. E. Riesgo Escudero, S. E. Riesgo, E. Zaballos Castellvi, M. Herranz Fernández, J. Alins Presas, I. Damas Pérez, I. Monte Collado, R. Genique Martínez, M. J. Guasch Villanueva, T. Rama Martínez, L. Pinto Pena, J. M. Panisello Royo, I. Gil Gil, E. Alarcón Cebrián, M. J. Piñero Acin, C. Pecharromán Sacristán, M. S. Mayayo Vicente, M. P. Pérez Unanua, N. Marañón Henrich, S. Gómez Monreal, S. Redondo De Pedro, B. Sanz Pozo, I. Moreno Martínez, M. Zuleta Isaza, B. López Uriarte, C. Sanz Velasco, A. Gárriz Aguirre, G. Reviriego Jaén, J. I. Aza Pascual-Salcedo, J. Vázquez Gallego, J. Caballer Rodilla, A. Herrera, E. Arranz Martínez, A. M. Gómez Calvo, P. Morán Oliva, M. M. González Béjar, J. A. Heras Hitos, O. García Vallejo, M. D. J. Frías Vargas, M. J. Castillejo Boguerin, A. García Lerín, M. Á. María Tablado, E. C. García García, M. E. Montes Belloso, A. M. Huertas Velasco, R. Sáez Jiménez, J. N. García Pascual, M. C. Díez Pérez, A. Ruiz García, C. Murillo Jelsbak, V. Lasso Oria, A. González Gamarra, M. Mestre De Juan, M. C. García Albiñana, P. Casado Pérez, J. Espinosa García, J. I. Prieto Romo, L. Fernández Fernández, J. Sierratapia, N. Moreno Regidor, F. J. Zaballos Sánchez, A. Moreno Moreno, F. Carramiñana Barrera, M. J. Gamero Samino, M. Á. De Santiago Rodríguez, A. C. Elías Becerra, J. Domínguez Ávila, A. S. Fuentes, J. M. De Nicolás Jiménez, D. Igual Fraile, G. Nieto Barco, I. Araujo Ramos, M. L. Serrano Berrocal, F. Buitrago Ramírez, M. Gallego Marcos, F. Suárez González, V. Chavero Carrasco, J. Polo García, F. Guerra Peguero, F. J. Sánchez Vega, M. Tejero Mas, A. Palmerín Donoso, M. Turégano Yedro, E. García Del Río, J. Álvarez Fernández, P. Alonso Álvarez, M. L. Jorge Gómez, A. Calvo Guerrero, L. Barreiro Casal, J. Fernández Moreno, M. A. Carballal Martínez, N. Díaz Rodríguez, C. Moral Paredes, D. Recarey García, A. Fouz Ulloa, N. Dios Parada, P. Conde Sabarís, A. I. Rodríguez Pérez, A. I. García Palacio, V. J. Quesada Varela, Á. Lado Llerena, C. Lires Rodríguez, M. L. Carretero Díaz, J. Carreira Arias, J. L. Vázquez Camino, M. D. C. Torreiro Penas, S. Yáñez Freire, S. Cinza Sanjurjo, D. Rey Aldana, C. Piñeiro Díaz, J. Rodríguez Campos, L. Vilela De Castro, S. M. Velilla Zancada, R. Crespo Sabarís, J. A. Benaín Ávila, Ó. Del Toro González, D. J. Rubira López, M. D. Esteve Franco, J. Castillo Meroño, J. M. Lobo Martínez, I. M. Peral Martínez, A. Santo González, J. Á. Rodríguez Calvillo, J. Gomáriz García, B. Ríos Morata, L. Sánchez Íñigo, V. Pascual Fuster, M. D. Aicart Bort, N. Vázquez Gómez, C. Lluna Gasco, T. Amorós Barber, P. A. Medina Cano, M. Monteagudo Moncho, R. Navarro Hernández, F. J. Martínez Egea, A. Tramontano, M. Ferrer Royo, B. Persiva Saura, J. A. Contreras Torres, A. Salanova Penalba, A. Cucó Alberola, F. M. Navarro I Ros, A. Seoane Novás, E. Peña Forcada, N. Aguilar Gómez, F. J. Sanz García, M. D. Paradís Bueso, M. E. Alegre Romero, A. Francés Camus, M. A. Antón Peinado, R. Latorre Santos, M. A. Palomar Marín, M. C. Botella García, E. Sánchez Fresquet, R. A. Valero Valero, M. Seoane Vicente, M. Martin Llinares, A. Masiá Alegre, J. L. Llisterri Caro, I. Lluch Verdú, V. Pallarés Carratalá, F. Valls Roca, R. M. Micó Pérez, E. Benages Vicente, M. J. Gimeno Tortajada, M. Menéndez Rodríguez. The team would also like to thank SEMERGEN.

**AEGIS**. F. Gude Sampedro, A. González Quintela, J. Sánchez Castro, M.C. Fernández Merino, V. García-Ciudad Young, J. Rodríguez García. The team would like to thank the study participants.

**DI@BET.ES-EUSKADI**. A. Cobo, A.M. Megido, T. González-Frutos, C. Gil, J. Rioja, J.O. Casanovas-Marsal.

**CARGENCORS.** A. Camps-Vilaró, L. Zacarías-Pons, J. Marrugat, R. Ramos Blanes, I.R. Dégano, R. Elosua, R. Martí Lluch, I. Subirana, S. Polo-Alonso, M. Pinsach-Abuin, M. Puigmulé, A. Pérez, X. Nogues, J.R. Masclans, R. Güerri-Fernández, J. Marin, H. Tizon-Marcos, B. Vaquerizo, R. Brugada. A full roster of contributors can be found at: <https://regicor.cat/cargencors_inv/>

**GCAT.** R. de Cid, J. Barretina, N. Blay, X. Farré, S. Iraola, V. Moreno, M. Obón-Santacana. The authors of the study would like to acknowledge all GCAT project investigators who contributed to generating the GCAT data, with a special mention to A. Carreras. A full list of the investigators is available at [www.genomesforlife.com](http://www.genomesforlife.com). We also thank Dr. J. Grifols on behalf of the Blood and Tissue Bank from Catalonia and all the GCAT volunteers who participated in the study. This study was conducted using data provided by the Catalan Agency for Quality and Health Assessment within the framework of the PADRIS Program.

**RICARTO**. G.C. Rodríguez Roca, A. Segura Fragoso, F.J. Alonso Moreno, A. Villarín Castro, L. Rodríguez Padial, J. Fernández Martín, J. Carrasco Flores, M.L. Rodríguez-García, M. Sánchez-Pérez.

**Appendix II.** Detailed funding sources of the individual CORDELIA cohorts.

**REGICOR**. European Commission: 20081312, 20121207. ISCIII: PMP22/00033, 96/0026-01, 96/1571, 99/9342, 01/0105-01, PI020471, CP05/00290, PI061254, PI081327, PS09/00456, PI11/01801, PI12/00232, PI15/00051, PI15/00064, PI18/00017, PI18/00030, PI21/00021, PI21/00040. Department of Health, Generalitat de Catalunya: SLT002/16/00088, SLT002/16/00145, SLT006/17/00234, SLT021/21/000015. AGAUR SGR 2021: 2021 SGR 00144. Fundació La Marató de TV3: 081630. RecerCaixa: RE087465. FBBVA: PR-16_BIO_CAR_0041. Centre d'innovació i desenvopament empresarial: RD08-1-0024. Agència d'informació, avaluació i qualitat en salut: 034/33/02. CIRIT: 1998TDOC 00041. Other: SAF2001-0431; MCYT; FSEACV C.ESP/EEUU; Spanish Society of Cardiology.

**ACRISC**. ISCIII: CM12/03287, CP1117/00012, PI14/00449.

**BARCOS**. ISCIII: FIS98/1952, PI06/0895, PI13/00116. RETIC-Red Temática de Investigación Cooperativa en Envejecimiento y Fragilidad (RD12/0043/0022). AGAUR SGR 2005: 2005SGR00762.

**AWHS**. Collaboration agreement signed by the Instituto Aragonés de Ciencias de la Salud (Government of Aragon, Spain) and Fundación Centro Nacional de Investigaciones Cardiovasculares (CNIC, ISCIII), and between General Motors España S.L. and the Government of Aragon (BOA nº 198, Nov 26, 2008). Collaboration agreement between the Department of Health, Social Wellbeing and Family (Government of Aragon, Spain) and Fundación Centro Nacional de Investigaciones Cardiovasculares (CNIC, ISCIII), for the continuation of the study between 2013-2018.

**SALMANTICOR**. ISCIII: PI14/00695, PI21/00369. Government of Castilla y León: GRS1030/A/14. Other: competitive grants and by the Spanish Cardiovascular

Network (RIC, CIBERCV), Spanish Ministry of Economy and Competitiveness, Obra Social “la Caixa” and Philips Ibérica Healthcare division.

**DI@BET.ES.** ISCIII: CIBER in Diabetes and Associated Metabolic Disorders–CIBERDEM, PI14/00710, PI14/01104, PI14/00970, PI14/00874, PIE14/00031, PI17/02136, PI20/01322.

**PIZARRA**. ISCIII: CIBER in Diabetes and Associated Metabolic Disorders–CIBERDEM, PI041883, PI051307, PI06/90564, PI-08/1592. Consejería de Economia, Innovacion, Ciencia y Empleo, Junta de Andalucía: P09-CTS-5125. Servicio Andaluz de Salud, Junta de Andalucia: 0124/2005, 0258/2007.

**PREDAPS**. The study was financed with funds from the group itself.

**DRECE**. ISCIII: PI08/90643, PI10/02123, PI14/01940. Fundación de Investigación Médica Mutua Madrileña: FIMM 2008/088. Instituto de Investigación Hospital 12 de Octubre: 2019/0047.

**ENRICA**. Non-conditional research contract Universidad Autónoma de Madrid-Sanofi-Aventis. ISCIII: PI08/0166, PI09/1626. Plan Nacional sobre Drogas: ND 2010/006.

**EPIC-Granada.** ISCIII: RTICC DR06/0020. Junta de Andalucía.

**EPIC-Gipuzkoa.** European Commission-FP7-HEALTH EC-GA: 279233. European Union (“Europe Against Cancer” program): SOC 93 102808 05E01, SOC 94 201988 05F01, SOC 95 200585 05F02, SOC 96 200.416, SOC 97 200302 05F02. ISCIII: 90CVV01066, 91CVV01296-0, 92CVV01061-0, PI020652, PI021834, PI 021598. Department of Health, Government of Basque Country. Scientific Foundation of Asociación Española Contra el Cáncer.

**EPIC-Murcia**. European Commission-FP7-HEALTH EC-GA: 279233. European Union (“Europe Against Cancer” program): SOC 97 200302 05F02. ISCIII: 92/0038, 95/0067, PI042342. Department of Health, Government of Murcia. Scientific Foundation of Asociación Española Contra el Cáncer. Scientific Foundation of Asociación Española Contra el Cáncer.

**EFRCV**. Murcia Regional Health Authority: BORM 11/01/1991. Spanish Health Research Fund: 92/0902, G03/065, PI052765. CIBERESP: AC07_010.

**MCC-Spain**. European Commission: FOOD-CT-2006-036224-HIWATE. Spanish Council of Ministers: Acción Transversal del Cancer (Oct 11, 2007). Instituto de Salud Carlos III-FEDER: CIBERESP-Acción estratégica 2009-2023, Red Temática de Investigación del Cáncer-RD12/0036/0036, PI08/1770, PI08/0533, PI08/1359, PS09/00773, PS09/01286, PS09/01903, PS09/02078, PS09/01662, PI11/01889, PI17CIII/00034. Fundación Marqués de Valdecilla: API 10/09. ICGC International Cancer Genome Consortium CLL: CLL-Genome Project. Junta de Castilla y León: LE22A10-2. Consejería de Salud, Junta de Andalucía: PI-0571-2009, PI-0306-2011, salud201200057018tra. Conselleria de Sanitat, Generalitat Valenciana: AP_061/10. RecerCaixa: 2010ACUP00310. Regional Government of the Basque Country. Consejería de Sanidad de la Región de Murcia. Spanish Association Against Cancer: GCTRA18022MORE. Catalan Government-AGAUR: 2014SGR647, 2014SGR850, 2017SGR723. Fundación Caja de Ahorros de Asturias. University of Oviedo.

**CORSAIB**. Health Department, Government of the Balearic Islands. ISCIII: FIS 02/1717, G03/065, PI 06/1228, RD06/0018/0045

**EMMA**. ISCIII: PI051936. Research Network on Chronicity, Primary Care, and Health Promotion (RICAPPS). RD21/0016/0001

**ARTPER**. ISCIII: PI07/90415, PI070403, PI11/00765, PI15/00510.

**NEFRONA**. ISCIII: PS10/0094. ABBOTT labs

**ILERVAS**. Diputació de Lleida. ISCIII: RETIC RD16/0009/0011, PI21/01099, PI23/00237 Spanish Ministry of Science, Innovation and Universities: IJC2018-037792-I.

**ASTURIAS**. ISCIII: 98/0462.

**CDC de Canarias**. ISCIII: 99/0361, PI021189, PI021158, PI070934, PS0901314, PI12/01855, RD12/0042/0031, PI18/01314. FUNCIS: 45/98, 36/00, 44/02, 66/04, 60/05. Department of Education, Government of the Canary Islands: PI042005/086, 2005/047. Agencia Española de Cooperación Internacional al Desarrollo: PCI 2006-A7-0648.

**HERMEX**. ISCIII: PI 071218, EMER 07/046, INT 07/289, 09/030, PI 14/00691, Red HERACLES-RD06/0009, CM08/00141. Government of Extremadura (Plan Integral Enfermedades Cardiovasculares de Extremadura, Programa de intensificación de la actividad investigadora en el Servicio Extremeño de Salud). Spanish Ministry of Education: FPU12/00963. European Union Marie Skłodowska-Curie actions (COFUND–Grant Agreement no. 291780),

**HORTEGA**. European Commission: Ingenious Hypercare (EPSS-037093). ISCIII: CP12/03080, PI15/00071, PI10/0082, PI13/01848, PI14/00874, PI16/01402, PI11/00726, CIBEROBN-CIBER-02-08-2009, CB06/03/101, CB12/03/30016, CIBERDEM. Government of Comunitat Valenciana: PROMETEO/2009/029, ACOMP/2013/039. Government of Castilla y Leon: GRS/279/A/08.

**NAVARRA 93**. The study was financed with funds from the group itself.

**EPIC-Navarra**. European Commission-FP7-HEALTH EC-GA: 279233. European Union (“Europe Against Cancer” program): SOC 97 200302 05F02. ISCIII: 99-0024.

**RIVANA**. Agreement between the ISCIII and the Department of Health of the Government of Navarra (Resolución 172/2004, Feb 20, 2004). Government of Navarra, government budgets from 2004 to 2010. Resoluciones 2004, 215/2005, 145/2006, 101/2007, 939/2008I, 754/2009 y 2010. ISCIII: PI05/2364.

**RECCYL**. Department of Health, Government of Castilla y León.

**PREDIMERC**. Government of the Madrid Region and ISCIII: PI07/1213.

**IBERICAN**. The study was financed with funds from the group itself.

**AEGIS**. ISCIII: PI11/02219, PI13/02594, PI16/01395, PI16/01404, PI20/01150.

**DI@BET.ES-EUSKADI**. Department of Health of the Government of the Basque Country: 2009COM11, GV2010111058, 2015111020. ISCIII: PI14/01104, CIBERDEM-CB07/08/0025. University of the Basque Country: IT795-13, IT1281-19. Endo-ERN: 739527.

**CARGENCORS.** Fundació La Marató de TV3: 2021/19-30. CRUE‐CSIC‐Santander Fondo Supera COVID–19.

**GCAT.** Spanish Ministry of Science & Innovation and the Catalan Ministry of Health of the Generalitat of Catalunya: “Dinamización Acción Program” (ADE 10/00026), the “GEPETO project” (TED2021-130626B-I00) and the “DATOS-CAT project”. ISCIII: PI18/01512. European Regional Development Fund (ERDF), Programa Operativo FEDER de Catalunya 2014-2020: 001-P-001647.

**RICARTO**. Government of Castilla-La Mancha: PI-2010/043. Fundación Sociosanitaria de Castilla–La Mancha (FISCAM). Non-directed fellowship of the Fundación de la Sociedad Española de Médicos de Atención Primaria.

**Appendix III.** Ethical approval information in the individual CORDELIA cohorts.

**REGICOR.** The REGICOR cohort and its individual sub-projects have been approved by the Ethics Committee of Hospital del Mar Research Institute (Barcelona, Spain). The code of the project and the sub-projects approved within REGICOR are: 2008/3046/I; 2008/3122/I; 2009/3504/I; 2010/3779/I; 2011/4309/I; 2012/4729/I; 2014/5746/I; 2015/6530/I; 2015/6206/I; 2015/6206/I; 2015/6199/I; 2016/7075/I; 2017/7281/I; 2017/7764/I; 2018/7927/I; 2018/7855/I; 2019/8678/I; 2019/8757/I; 2021/9991/I; 2023/10785/I.

**ACRISC.** The project was approved by the Ethics Committee of Hospital del Mar Research Institute (Barcelona, Spain). The code of the project is: 2014/581517.

**BARCOS.** The project was approved by the Ethics Committee of Hospital del Mar Research Institute (Barcelona, Spain).

**AWHS**. The project was approved by the Clinical Research Ethics Committee of Aragon (Comité Ético de Investigación Clínica de Aragón, CEICA. The project code is PI07/09, date: May 16, 2007.

**SALMANTICOR**. The project was approved by the Clinical Research Ethics Committee of the health area of Salamanca (CEIC del Área de Salud de Salamanca), date: 29 Sep, 2014.

**DI@BET.ES.** The project was approved by the Clinical Research Ethics Committee of Malaga (reference 21/02/2019) and of Hospital Universitario Carlos Haya (reference SE-037).

**PIZARRA.** The project was approved by the Clinical Research Ethics Committee of Malaga (reference 21/02/2019) and the Comision permanente del CEI Málaga Nordeste (reference 13/02/2012).

**PREDAPS.** The project was approved by the Ethics Committee of Hospital del Mar Research Institute (2011 Barcelona, Spain).

**DRECE**. The DRECE cohort and its sub-projects have been approved by the Clinical Research Ethics Committee of Hospital Universitario 12 de Octubre (references: 10/292, 14/370, 19/116).

**ENRICA**. The project was approved by the Clinical Research Ethics Committee of Hospital Clínico de Barcelona (reference 2007/3544) and Hospital Universitario La Paz in Madrid (reference HULP 2144).

**EPIC-Granada.** The EPIC project was approved by the ethical review boards from the International Agency for Research on Cancer (IARC) (Lyon, France) as documented in the "Minutes of the Meeting of the IARC Ethical Review Committee held in the President's Lounge on 12 January 1995.". The approval was ratified by the IARC Ethics Committee on May 10, 2017.

**EPIC-Gipuzkoa.** The EPIC project was approved by the ethical review boards from the International Agency for Research on Cancer (IARC) (Lyon, France) as documented in the "Minutes of the Meeting of the IARC Ethical Review Committee held in the President's Lounge on 12 January 1995.". The approval was ratified by the IARC Ethics Committee on May 10, 2017.

**EPIC-Murcia.** The EPIC project was approved by the ethical review boards from the International Agency for Research on Cancer (IARC) (Lyon, France) as documented in the "Minutes of the Meeting of the IARC Ethical Review Committee held in the President's Lounge on 12 January 1995.". The approval was ratified by the IARC Ethics Committee on May 10, 2017.

**EFRCV**. The study was conducted with the approval of the Ethics Committee of the Virgen de la Arrixaca Hospital (Murcia).

**MCC-Spain**. The project was approved by the Clinical Research Ethics Committees of the following institutions: CEIC de Navarra (date: Sep 25, 2008), CEIC de la DGSP y CSISP de Valencia (date: Jul 14, 2010), Instituto Municipal de Asistencia Sanitaria (date: Sep 2, 2008), Hospital General Universitario Jose María Morales Meseguer de Murcia (date: Nov 12, 2008), Hospital Universitario Juan Ramón Jiménez de Huelva, Hospital Universitario de Doctor Josep Trueta de Girona, Hospital Universitario de la Paz (date: Feb 5, 2009), Hospital Ramón y Cajal (date: Nov 4, 2008), CEIC de Cantabria (date: May 8, 2009), Hospital Clinic de Barcelona (date: Jul 10, 2008), CEIC regional del Principado de Asturias (date: Oct 17, 2008), CEIC de Euskadi (date: Sep 25, 2008), and CEIC de León (date: Jan 27, 2009).

**CORSAIB**. The project was approved by the Clinical Research Ethics Committee of the Balearic Islands (reference: IB1999).

**EMMA.** The project was approved by the Clinical Research Ethics Committee of the IDIAP Jordi Gol.

**ARTPER.** The project was approved by the Clinical Research Ethics Committee of the IDIAP Jordi Gol (reference: P06/64 - 2006/12/20).

**NEFRONA.** The project was approved by the Clinical Research Ethics Committee of the Hospital Universitario Arnau de Vilanova in Lleida (reference: 13/2010).

**ILERVAS.** The project was approved by the Clinical Research Ethics Committee of the Hospital Universitario Arnau de Vilanova in Lleida (reference: 19/2014).

**ASTURIAS**. The project was approved by the Ethics Committee of the Health Service of the Principality of Asturias (1998)

**CDC de Canarias.** The project was approved by the Clinical Research Ethics Committee of the Hospital Universitario Nuestra Señora de Candelaria in Tenerife.

**HERMEX.** The project was approved by the Clinical Research Ethics Committee of the Hospital Regional Universitario Infanta Cristina in Badajoz (date; Jun 29, 2005).

**HORTEGA.** The project was approved by the Clinical Research Ethics Committee of the Hospital Universitario Río Hortega in Valladolid (date: Jan 12, 2003).

**NAVARRA 93.** The project was approved by the Clinical Research Ethics Committee of Navarra (reference: PI_2019/79, date: Aug 28, 2019).

**EPIC-Navarra.** The EPIC project was approved by the ethical review boards from the International Agency for Research on Cancer (IARC) (Lyon, France) as documented in the "Minutes of the Meeting of the IARC Ethical Review Committee held in the President's Lounge on 12 January 1995.". The approval was ratified by the IARC Ethics Committee on May 10, 2017.

**RIVANA.** The study and the cohort sub-projects were approved by the Clinical Research Ethics Committee of Navarra (reference: PI 4/04, date: May 11, 2004; reference: PI 1/05, date: Jun 21, 2005; reference: PI 2018/91, date: NOV 23, 2018 and reference PI 2020/108, date SEP 28, 2020).

**RECCYL**. The Clinical Research Ethics Committee of the Rio Hortega University Hospital approved the 10 years follow up research project of the RECCYL Cohort in 2013.

**PREDIMERC.** The project was approved by the Clinical Research Ethics Committee of Hospital Ramón y Cajal (date: Feb 9, 2007, reference 240/06).

**IBERICAN.** The project was approved by the Clinical Research Ethics Committee of Hospital Clínico San Carlos in Madrid (reference: 13/047-E, date: Feb 21, 2013).

**AEGIS**. The cohort and its sub-projects were approved by the Clinical Research Ethics Committees of Galicia (reference: 2012/025, date: April 26, 2012) and Santiago-Lugo (reference: 2016/240, date: May 24, 2016; reference: 2021/250, date: May 25, 2021).

**DI@BET.ES-EUSKADI**. The project was approved by the Clinical Research Ethics Committee of Euskadi (reference: PI2016042).

**CARGENCORS.** The project was approved by the Ethics Committees of Parc de Salut Mar (reference: 2020/9297/I, date: July 9, 2020; reference: 2020/9650/I, date: May 12, 2021), Doctor Josep Trueta University Hospital of Girona (reference: 2020/058, date: May 11,2020), and Foundation University Institute for Primary Health Care Research Jordi Gol i Gurina (reference: 2021/084‐PCV).

**GCAT.** The project was approved by the Clinical Research Ethics Committee Germans Trias i Pujol (reference: PI-13-020, date: April 26, 2013).

**RICARTO**. The project was approved by the Clinical Research Ethics Committee of Hospital Virgen de la Salud-Complejo Hospitalario de Toledo (date: Dec 9, 2009).
